# Flexible Gates Generate Occluded Intermediates in the Transport Cycle of LacY^☆^

**DOI:** 10.1016/j.jmb.2013.10.024

**Published:** 2014-02-06

**Authors:** Lukas S. Stelzl, Philip W. Fowler, Mark S.P. Sansom, Oliver Beckstein

**Affiliations:** 1 Department of Biochemistry, University of Oxford, South Parks Road, Oxford OX1 3QU, UK; 2 Center for Biological Physics, Department of Physics, Arizona State University, Tempe, AZ 85287, USA

**Keywords:** DEER, double electron–electron spin resonance, DIMS, dynamic importance sampling, MD, molecular dynamics, MFS, major facilitator superfamily, MFS, major facilitator superfamily, POPC, 1-palmitoyl,2-oleoyl-*sn*-glycero-3-phosphocholine, SGLD, self-guided Langevin dynamics, 3D, three-dimensional, major facilitator superfamily transporters, molecular dynamics simulations, protein conformational change, membrane permeation, protons

## Abstract

The major facilitator superfamily (MFS) transporter lactose permease (LacY) alternates between cytoplasmic and periplasmic open conformations to co-transport a sugar molecule together with a proton across the plasma membrane. Indirect experimental evidence suggested the existence of an occluded transition intermediate of LacY, which would prevent leaking of the proton gradient. As no experimental structure is known, the conformational transition is not fully understood in atomic detail. We simulated transition events from a cytoplasmic open conformation to a periplasmic open conformation with the dynamic importance sampling molecular dynamics method and observed occluded intermediates. Analysis of water permeation pathways and the electrostatic free-energy landscape of a solvated proton indicated that the occluded state contains a solvated central cavity inaccessible from either side of the membrane. We propose a pair of geometric order parameters that capture the state of the pathway through the MFS transporters as shown by a survey of available crystal structures and models. We present a model for the occluded state of apo-LacY, which is similar to the occluded crystal structures of the MFS transporters EmrD, PepT_So_, NarU, PiPT and XylE. Our simulations are consistent with experimental double electron spin–spin distance measurements that have been interpreted to show occluded conformations. During the simulations, a salt bridge that has been postulated to be involved in driving the conformational transition formed. Our results argue against a simple rigid-body domain motion as implied by a strict “rocker-switch mechanism” and instead hint at an intricate coupling between two flexible gates.

## Highlights

• The transport mechanism of LacY is hypothesized to involve an intermediate “occluded” state.
• Such a state is observed in computer simulations of the conformational transitions.
• Simulation data are validated with experimental double electron–electron spin resonance measurements.
• The structural gating elements of LacY are identified.
• Occluded LacY is similar to known occluded structures of homologous proteins.

## Graphical abstract

**Figure:**
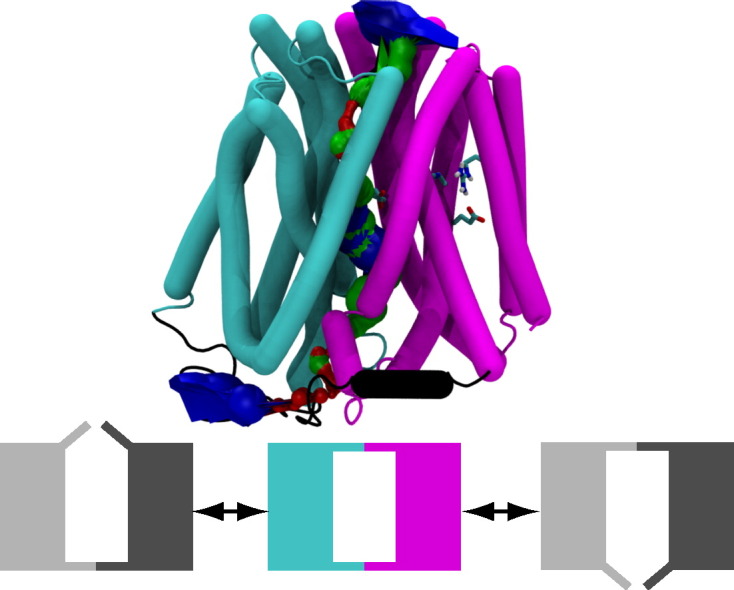

## Introduction

Lactose permease (LacY) is the best-studied member of the major facilitator superfamily (MFS) of secondary transporters [1]. LacY of *Escherichia coli* is a symporter and uses the proton (H^+^) electrochemical gradient to transport galactosides, such as lactose, from the periplasm into the cytoplasm [2]. The structural elements that allow or prevent exchange of sugar and H^+^ between the central binding site and either the cytoplasm or the periplasm are often referred to as “gates” [3]. LacY transports substrate by switching from a conformation with an open cytoplasmic gate and a closed periplasmic gate to a periplasmic open conformation in which the states of the gates are reversed. This alternating access mechanism [4], [5] has been detected [6] in site-directed alkylation [7] and cross-linking [8], tryptophan quenching [9], single-molecule Förster resonance energy transfer [10] and double electron–electron spin resonance (DEER) [11] experiments.

The transition between the cytoplasmic and periplasmic open states of LacY is not understood in full atomic detail. Partly, this reflects a lack of structural data. Only crystal structures for cytoplasmic open [12], [13], [14], [15] LacY have been obtained. The crystal structure of the distantly related MFS transporter FucP from *E*. *coli* has been solved in a periplasmic open conformation [16]. Radestock and Forrest have built two models of periplasmic open LacY: the first is based on its homology to the FucP structure and the second uses the internal inverted repeat symmetry of the transporter [17]. Biochemical studies [2] have identified key events in the transport. First, cytoplasmic open LacY releases the transported sugar to the cytosol. The subsequent deprotonation of E325 initiates the transition to the periplasmic open conformation [18]. Periplasmic open LacY is a minor conformer [2] in the absence of substrate. Protonation of H322 and substrate binding then stabilizes the periplasmic open state and initiates the return to the cytoplasmic open conformation, thereby carrying sugar and H^+^ across the membrane.

**Fig. 1:**
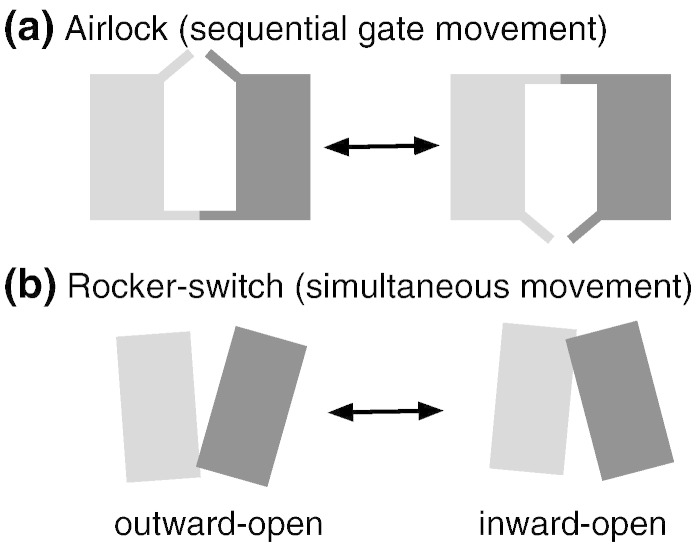
Schematic models of transport resulting in alternating access. A transporter consisting of two domains (light and dark gray) may undergo conformational transitions that expose a central substrate binding site alternatively to the extracellular and the intracellular side. (a) In the *airlock model*, two gates open and close sequentially to form an outward open and inward open conformation. In order to prevent formation of a leak state, the gates must be strongly coupled so that they never open at the same time. (b) In the *rocker-switch model*, the two domains move simultaneously as rigid bodies relative to each other and thus form the outward open and inward open states. No additional coupling is required to prevent formation of a leaky transporter.

In addition to biochemical evidence, functional considerations provide some clues about the mechanism of the conformational transition. A transporter with both gates open would dissipate vital transmembrane gradients, such as the proton gradient in bacteria or the sodium gradient in higher eukaryotes to the detriment of the cell that has expended ~ 20% of its energy to establish the gradient [19]. The two gates of a transporter should therefore never be open at the same time [3] and it is thought that, to prevent this, the movement of the gates must occur in a precise order so that one gate closes before the other starts to open as in the operation of an airlock (Fig. 1a) [20]. Such an “airlock model” for transport predicts an occluded intermediate conformation that is closed to ion and substrate permeation from both cytoplasm and periplasm. The occluded conformation is thus fundamental to the transport mechanism. Preventing both gates from being open at the same time necessitates coupling between the movements of the two gates so that only the closure of one gate allows opening of the other gate. We describe the case where one gate must close fully before the other can begin to open as *a sequential motion*. In this case, the transporter passes through intermediates that are fully occluded.

It has been hypothesized that a rigid-body movement of the N- and C-terminal domains of LacY relative to one another [16] (Fig. 1b) would allow the cytoplasmic and periplasmic open states to interconvert. This model is called the “rocker-switch mechanism” [1], [12]. The rigidity of the domains would provide a direct mechanical linkage between the two gates so that the closure of one gate would occur simultaneously with the opening of the other. We describe this as *a simultaneous motion*. A truly rigid motion of the two domains would probably not be able to form a fully occluded conformation. Allowing the domains some flexibility would allow the formation of an occluded conformation, ruling out the dissipation of transmembrane potentials through channel-like conformations. Hydrogen/deuterium exchange experiments have established that LacY is more dynamic than many other membrane proteins [21], [22], [23] and is therefore unlikely to contain large, rigid domains. The conformational flexibility of LacY has also been highlighted in NMR experiments [24] and in molecular dynamics (MD) simulations [24], [25].

In the absence of direct evidence, we cannot be certain if LacY passes through an occluded intermediate and, if it does, what such an intermediate would look like. DEER (also known as PELDOR) measurements for a range of residue pairs on LacY have been interpreted to demonstrate the existence of an intermediate occluded state [11], [26] and have recently been used to propose a model of an occluded conformation of LacY [27]. To date, occluded conformations of five MFS transporters at atomic resolution have been published. These are EmrD [28], NarU [29], XylE [30], [31] (all from *E*. *coli*), PepT_So_ [32] (from *Shewanella oneidensis*) and PiPT [33] (from the fungus *Piriformospora indica*). In addition, an occluded conformation of the MFS transporter OxlT from *Oxalobacter formigenes* was resolved by electron microscopy, albeit at a lower resolution of 6.5 Å [34].

Computer simulations can suggest intermediate conformations and plausible transition mechanisms [35] that may then be compared to experimental data. The crystal structures of LacY and GlpT transporters are in cytoplasmic open conformations. The cytoplasmic gate of both structures has been observed to undergo partial closure in equilibrium MD simulations, thereby forming partially occluded conformations [18], [25], [36]. The turnover number for LacY is 16–21 s^− 1^ [37], thus, on average, one transition every 50,000 μs would be expected. At present, standard atomic-resolution equilibrium MD simulations typically yield trajectories of the length of up to ~ 1 μs and hence we cannot readily characterize the kinetics of transport using such simulations. Reducing the level of detail modeled is one way to overcome this challenge and this approach enabled the transition between cytoplasmic and periplasmic open FucP to be studied using an elastic network model [38], which hinted at the existence of an occluded intermediate. Alternatively, fully atomistic approaches that bias the sampling, such as stratified umbrella sampling [39], dynamic importance sampling (DIMS) MD [40], [41], [42] or self-guided Langevin dynamics (SGLD) [43], can be used to study such conformational changes. Pendse et al. used SGLD to simulate the transition from cytoplasmic open to periplasmic open LacY [44]. Although transition intermediates obtained from the SGLD simulations contained both cytoplasmic and periplasmic constrictions, no detailed study of the likely functional relevance of these structures was presented.

**Fig. 2:**
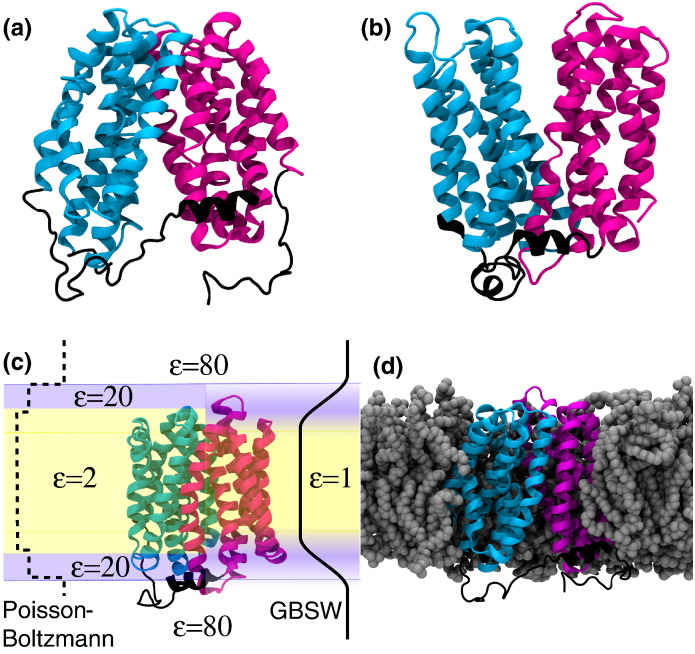
Atomistic simulations of LacY. The wild-type crystal structure of cytoplasmic open LacY (PDB code 28VN) (a) [14] and a FucP-based homology model for periplasmic open LacY (PMDB PM0077183) (b) [17] used in atomistic simulations are shown with the periplasmic face at the top and the cytoplasmic face at the bottom. The N-terminal and C-terminal domains, residues 7–185 and 220–399, are colored cyan and magenta, respectively. The linkers connecting the domains and flexible N- and C-terminal stretches are shown in black. (c) The membrane and solvent environment was represented implicitly as regions with differing dielectric constants ε in electrostatics calculations (using the Poisson-Boltzmann approach [45]) and simulations of rare events (with the GBSW solvent model [46], [47], [48]). (d) In equilibrium MD simulations, both the protein and the membrane and solvent environment were represented in full atomistic detail. Cytoplasmic open LacY inserted into a POPC membrane, highlighted in gray, is shown here. The solvent molecules and ions are omitted for clarity. The structures were rendered in VMD [49].

In this paper, we will investigate if LacY switches conformation via an occluded state and what conformation the protein could plausibly adopt in such a state. These results will then allow us to analyze if the broad conformational changes involved in the transport mechanism are compatible with either the airlock or the rocker-switch mechanism. We will use both equilibrium and DIMS [40], [41] MD simulations to study transitions between the cytoplasmic open and periplasmic open conformations of LacY. In particular, we will use the biased DIMS MD simulations in implicit solvent to accelerate sampling of conformational transitions and then study conformations in full detail with explicit solvent equilibrium MD (Fig. 2), similar to the approach taken by Pendse et al. [44]. The simulations reveal fully occluded intermediate structures. By introducing geometric order parameters that track the states of the two gates, we show that our results are consistent with crystal structures of related MFS transporters. Furthermore, our simulations also agree with experimental DEER measurements [11] of LacY and exhibit the formation of a salt bridge postulated to be involved in driving the conformational transition [15], [18].

## Results and Discussion

### Equilibrium MD simulations of cytoplasmic open and periplasmic open conformations

**Fig. 3:**
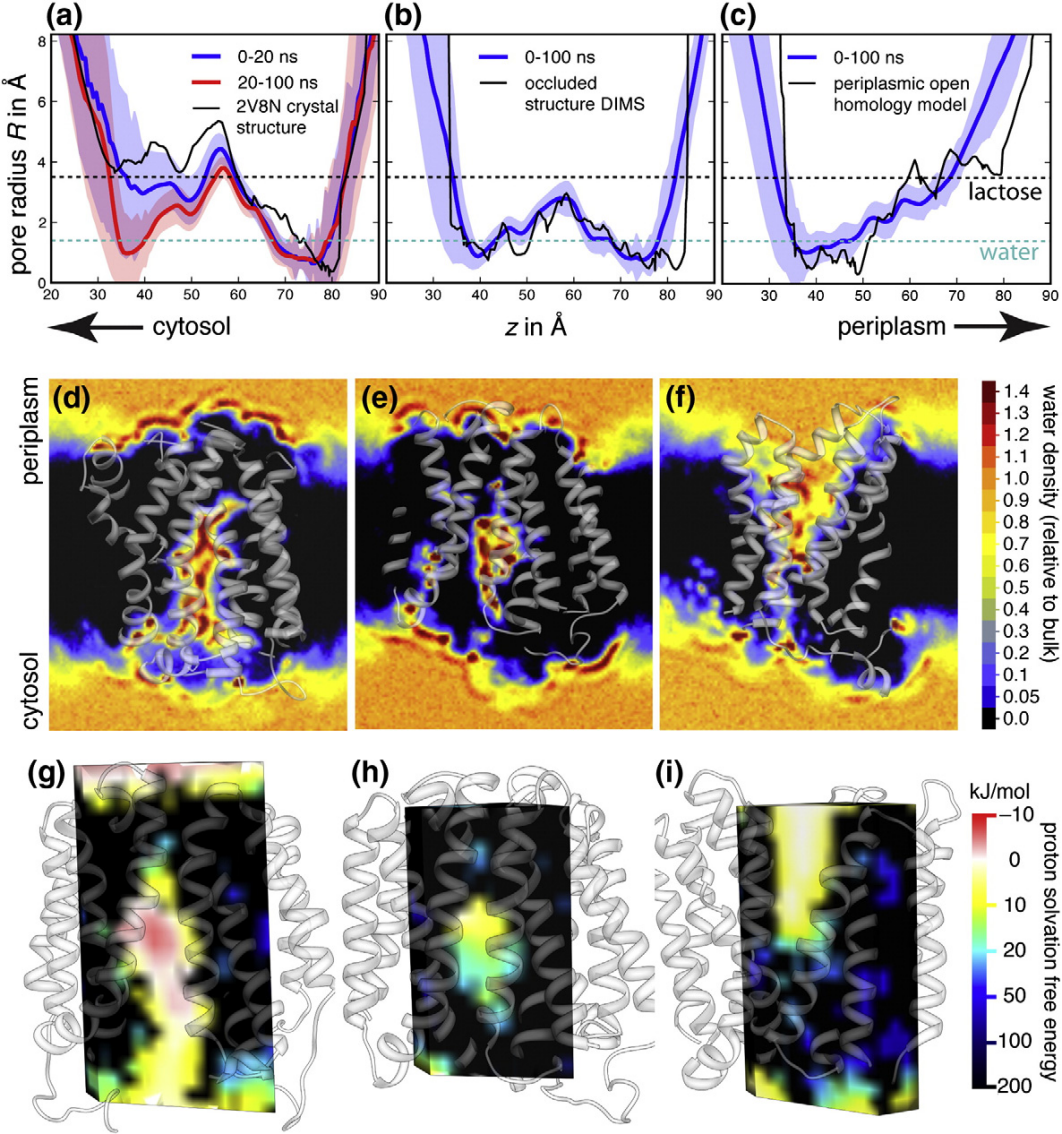
Functional characterization of LacY in MD simulations of multiple conformational states. Pore radii as determined by HOLE [50] are shown along the membrane normal *z* for the central cavity in simulations cytoplasmic open *COpenA1* (a), occluded *Occ* (b) and periplasmic open *POpenA* (c). Shaded regions indicate 1 SD of the radius from the mean value. Water densities relative to bulk SPC water [51] (0.970 g/cm^3^) were calculated with gridcount [52], [53] and visualized as 3D densities in UCSF Chimera [54], [55]. Cuts through the densities for simulations *COpenA1* (d), *Occ* (e) and *POpenA* (f) are shown. For *COpenA1* and *Occ*, the last frame from 100 ns of equilibrium MD is depicted. For *POpenB*, the equilibrated starting structure for the 100-ns simulation is shown. The position-resolved electrostatic free energy *W*_elec_(*r*) for solvated H^+^ interacting with cytoplasmic open (g), fully occluded (h) and periplasmic open LacY (i), calculated with BornBrofiler [45] (see Materials and Methods) and visualized in UCSF Chimera [54], [55] is shown.

We first simulated both end states of the conformational cycle in a 1-palmitoyl,2-oleoyl-*sn*-glycero-3-phosphocholine (POPC) membrane (Fig. 2) to probe the conformational flexibility (discussed in further detail in the supplementary information, especially in Figs. S1 and S2) of the transporter and establish a baseline for its dynamical behavior. The wild-type crystal structure of LacY [14] served as the cytoplasmic open conformation (see Table 1 for a list of all simulations). For the periplasmic open conformation, we used Radestock and Forrest's homology model [17], which is based on the periplasmic open FucP structure [16]. We simulated the apo conformation of both end states; that is, there was no sugar bound and E325 was not protonated. The apo conformations should be on the cusp of changing conformation [2] and thus simulation might reveal the initial stages of the transition. Since simple visual inspection of a static structure is not sufficiently accurate to determine if the central binding site is accessible to both substrate and protons, we will apply a range of computational methods to assess the functional state of the generated protein conformations (Fig. 3). We will first characterize the functional state of the transporter in the simulations (i.e., which gates are open or closed) by calculating the radius of the translocation pathway through the transporter. Then, we shall examine the density of water and the electrostatic free energy of a permeating H^+^. These are more sensitive and together will allow us to assign the likely functional state of any structure.

**Table 1:** Overview of the equilibrium simulations of LacY.

Name of simulation	Starting structure	Number of POPC lipids	Length of simulation (ns)	Force field
*COpenA1*	2V8N crystal structure	126	100	GROMOS96 43a1
*COpenA2*	*COpenA1* end structure	126	100	GROMOS96 43a1
*COpenB1*	2V8N crystal structure	256	100	GROMOS96 43a1
*COpenB2*	*COpenB1* end structure	256	200	GROMOS96 43a1
*COpenC*	2V8N crystal structure	152	100	CHARMM22 + CHARMM36
*POpenA*	FucP-based homology model	254	100	GROMOS96 43a1
*POpenB*	FucP-based homology model	254	100	CHARMM22 + CHARMM36
*Occ*	End structure DIMS cytoplasmic closure	256	100	GROMOS96 43a1
*COpen-GBSW*	2V8N crystal structure	Implicit membrane	10	CHARMM22 + GBSW
*POpen-GBSW*	FucP-based homology model	Implicit membrane	~ 12	CHARMM22 + GBSW
*Occ-GBSW*	End structure of *Occ*	Implicit membrane	10	CHARMM22 + GBSW

During equilibrium MD simulations of the model of periplasmic open LacY, the periplasmic gate remained open and the cytoplasmic gate stayed closed (see simulation *POpenA* in Fig. 3c and repeat simulation *POpenB* in the supplementary content). These simulations demonstrated that both the conformation and its functional state were maintained over the duration of the simulations.

In contrast, during simulations of cytoplasmic open LacY, the periplasmic gate remained closed but the cytoplasmic gate started to close. In simulation *COpenA1*, the minimum radius on the cytoplasmic side of the transporter decreased from ~ 4 Å in the crystal structure to ~ 1 Å (Fig. 3a). The same effect was seen in multiple simulations with different box dimensions and force fields (as evidenced by the pore radius profiles in Fig. S3). The observed closure did not therefore depend on the dimensions of the box or the force field used. The variation of pore radius along the membrane normal suggests that sugars would be sterically blocked from entering the central cavity from both cytoplasm and periplasm. Our results are consistent with earlier studies that observed a similar closure of the cytoplasmic gate in multiple equilibrium MD simulations [18], [25] or a more limited closure after 10 ns of simulation [56]. Taken together, these results show that the cytoplasmic open apo structure of LacY, as resolved by X-ray crystallography, quickly relaxes to a more occluded structure that would prevent sugar permeation and might be a functionally relevant intermediate [25].

We assessed if this intermediate is functionally occluded, that is, whether solvated protons could still permeate, despite the closure of the cytoplasmic gate. We first calculated water densities from the MD simulations to define aqueous pathways that could act as proton conduits. Since the desolvation of a charged species is energetically unfavorable (in the absence of compensating interactions), dehydration of a gate will prevent ion permeation [45], [52]. The water density for the simulation of cytoplasmic open LacY (Fig. 3d and Fig. S4a and b) indicated that the constriction between the cytoplasm and the central cavity remained partially solvated even after the initial closure of the cytoplasmic gate, indicating that protons could still be expected to permeate and thus the conformation cannot be considered a functionally occluded state. No solvent-filled pathway connected the cavity and the periplasm in the simulation of cytoplasmic open LacY and thus the periplasmic gate remained completely shut. The cytoplasmic gate in the simulations of periplasmic open LacY remained dehydrated (Fig. 3f) whereas a wide aqueous pathway connected the central cavity to the periplasmic solution, suggesting the conformation represents a functional periplasmic open state.

Poisson–Boltzmann calculations of the electrostatic free energy of a solvated proton offer another way of assessing whether a gate is open since the heights of the energetic barriers between the cavity and bulk can be quantified [45]. The energies calculated for the cytoplasmic open (Fig. 3g) and periplasmic open (Fig. 3i) conformations suggest that the cytoplasmic and the periplasmic gates, respectively, offer no barrier to the movement of protons and thus would permit relatively free exchange of H^+^ between the bulk solvent and the central cavity. Electrostatic free energies in the cytoplasmic and periplasmic vestibules and the central cavity were only slightly less favorable than bulk (0–10 kJ/mol). The periplasmic gate of cytoplasmic open LacY is closed because H^+^ exchange between the central cavity and the periplasm would involve crossing a large volume with unfavorable electrostatic free energies (> 100 kJ/mol; Fig. 3g). By the same reasoning, the cytoplasmic gate of the periplasmic open transporter is also closed to H^+^ permeation (Fig. 3i). The initial closure of the cytoplasmic gate seen in Fig. 3a led to only a limited increase in the electrostatic free-energy barrier in the space connecting the cavity to the cytoplasm, from 0–10 kJ/mol to 10–20 kJ/mol (Fig. S5). This modest increase in the electrostatic free energy for proton permeation together with the water density calculations suggests that the observed closure of the cytoplasmic gate resulted in a partially functionally occluded intermediate. The gate appears therefore impermeable to sugar but not to H^+^ exchange between the cytoplasm and the internal cavity.

### DIMS simulations of gate closure yield a fully occluded conformation

Eight equilibrium MD simulations of cytoplasmic and periplasmic open LacY with an aggregated total time of 0.8 μs yielded neither a transition between the end states nor a functionally fully occluded intermediate. This is not surprising given that, on average, one transition would be expected to be observed every 50,000 μs for a typical turnover number of 16–21 s^− 1^ [37]. We therefore used DIMS MD [40] to investigate whether LacY passes through an occluded state as it changes from a cytoplasmic open conformation to a periplasmic open conformation. In DIMS MD, an importance sampling procedure is used to generate transitions between an initial structure and a target structure. DIMS MD has been successfully applied to study how other membrane proteins, such as a potassium ion channel [57] and a transporter [58], change conformation.

We first used DIMS MD to generate trajectories starting from the cytoplasmic open structure of LacY during which the cytoplasmic gate would close (these will be referred to as *DIMS-closure*). It was assumed that the cytoplasmic and periplasmic gates are located on the cytoplasmic and periplasmic sides of the transporter, respectively. Therefore, only the cytoplasmic half of periplasmic open LacY, that is, the one featuring the closed cytoplasmic gate, was used as the target structure. The DIMS importance sampling procedure then used this target structure to select pathways that led to cytoplasmic closure. The variation in the pore radius profile along the membrane normal of the final structure from the DIMS MD simulation showed constrictions on both the cytoplasmic and periplasmic sides (Fig. 3b), consistent with it being an occluded structure. A similarly constricted structure was generated from periplasmic open LacY using the periplasmic half of cytoplasmic open LacY, that is, the closed periplasmic gate, as the target structure (data not shown).

To help assess whether the DIMS method had produced a functionally fully occluded conformation, we simulated the final structure from DIMS MD for 100 ns (simulation *Occ*) using standard equilibrium MD. The putative occluded conformation was stable for the entire simulation as shown by the RMSD relative to the starting conformation (Fig. S1d) and the preservation of the secondary structure (Fig. S2c). The central water-filled internal cavity kept its shape. We then calculated how the pore radius varied along the membrane normal during the trajectory *Occ* (Fig. 3b). These pore profiles show that the minimum pore radii at the cytoplasmic and periplasmic constrictions remained smaller than the radius of a water molecule (~ 1.4 Å) during the entire simulation. The radius of gyration of lactose is 3.5 Å and therefore sugar substrates would not be able to access the central cavity. The pore profile is similar to the one observed in MD simulations of the occluded structure of the related MFS transporter EmrD [59].

The water density computed from the simulation of the occluded structure, *Occ*, was also consistent with both gates being closed. Slices through the three-dimensional (3D) water density show that regions with very low density separate the central cavity from both the cytoplasm and the periplasm. The internal cavity remained solvated (Fig. 3e) at between 0.7 and 1.4 times the bulk water density. Some water, however, remained in the interfacial regions. For example, on the cytoplasmic side, water density at 70% of bulk water is apparent in the slice shown in Fig. 3e but only regions with low density connect it to the cytoplasm and the internal cavity. While ions are likely to be stable in the water-filled central cavity, there are no continuous permeation pathways joining either the periplasm or the cytoplasm to the central cavity.

**Fig. 4:**
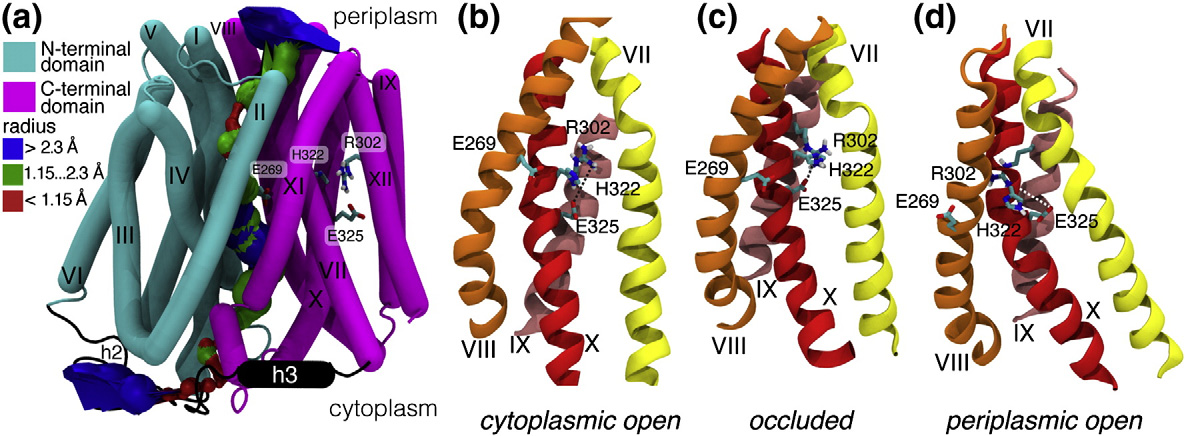
Occluded state model. (a) Helix representation of the occluded state model from the end of the *Occ* equilibrium simulation (rendered with Bendix [60] and VMD [49]). The N- and C-terminal domain are color-coded. The internal surface of the cavity as calculated by HOLE is shown. Residues essential for conformational changes and transport are shown as sticks. (b) Close-up of residues E269, R302, H322 and E325 in the cytoplasmic open conformation. The average distance of R302–E325 (broken line) is 5.1 ± 0.6 Å (mean ± SD) over the course of the equilibrium MD simulation. (c) The average R302–E325 distance is 3.3 ± 0.5 Å in the occluded state. (d) The FucP-based homology model of the periplasmic state places R302 and E325 far apart and they remain separated over the MD simulation (14.0 ± 0.9 Å). The time course of the interaction between E325 and R302 is shown in the supplementary information (Fig. S6) for simulations *COpenA1*, *POpenA* and *Occ*.

The structures generated by the *Occ* simulation were clustered (see Materials and Methods). The last frame of the simulation was chosen as a representative structure (Fig. 4a) since it belonged to the dominant cluster. This structure is of comparable quality to the wild-type crystal structure of cytoplasmic open LacY (see the supplementary content). The electrostatic free energy of placing a proton in this structure was calculated as before. Energies in the internal cavity ranged from 0 kJ/mol to 20 kJ/mol (Fig. 3h), but importantly, very high electrostatic free energies (> 100 kJ/mol) were obtained in the spaces on the periplasmic and cytoplasmic sides of the internal cavity. Some regions with more favorable electrostatic free energies (20–50 kJ/mol) exist between the internal cavity and the cytoplasm and the periplasm, as can be seen on the periplasmic side of the slice through the 3D density. Given that such regions were surrounded by space very unfavorable to protons, it is reasonable to conclude that electrostatic barriers to ion permeation from both the cytoplasm and the periplasm exist. The electrostatics and water density calculations are thus consistent with this structure of the transporter being not only sterically closed to sugar substrates but also occluded to protons and thus preventing a proton leak across the membrane.

Moreover, the model for the occluded state (Fig. 4a) is also consistent with the proposed mechanism for the transport cycle [2]. The residues crucial for function E269, R302, H322 and E325 (highlighted in Fig. 4), as well as E126 and R144 (data not shown), surround the central cavity as expected. In particular, we found that E325 and R302 form a stable salt bridge in simulation *Occ* and in our occluded state model (Fig. 4c) but neither in the cytoplasmic (Fig. 4b) nor in the periplasmic open (Fig. 4d) structures that were the inputs for our study. The two residues approach each other during the *DIMS-closure* simulation but the salt bridge only fully forms during the *Occ* equilibrium simulations as shown in Fig. S6 and discussed in more detail in the supplementary content. Experiments on an R302C/E325C mutant using excimer fluorescence suggested that these residues could be in close proximity [61]. Functional assays of mutants indicated that the formation of a salt bridge between R302 and E325 enables the release of the transported proton [62] so that the apo protein could transition from a cytoplasmic open to a periplasmic open conformation [18]. Thus, our computer simulation reveals a necessary step in the transport cycle and captures details of the molecular interactions that are hard to discern from static structures alone.

### Comparison of equilibrium MD simulations with DEER measurements

**Fig. 5:**
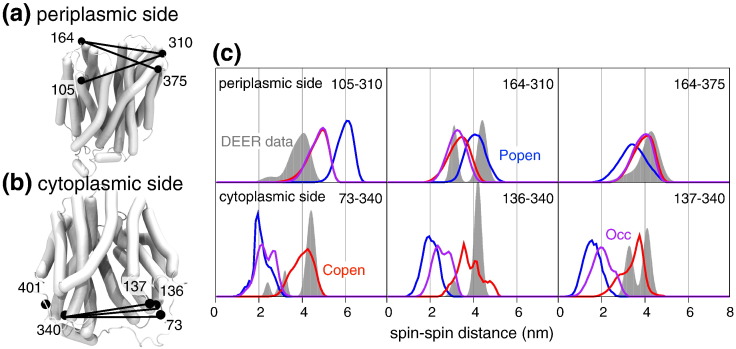
Experimental and simulated spin–spin distance distributions. (a) Location of spin labels on residues (black spheres, C^α^ atoms) and experimentally measured distances (black lines) on the periplasmic face of LacY (periplasmic open conformation). (b) Spin–spin distances on the cytoplasmic face (cytoplasmic open conformation). (c) Comparison of experimental DEER spin–spin distributions [11] (gray histograms, “DEER data”) to computed distributions from equilibrium MD simulations of LacY in three different conformational states: periplasmic open (blue lines, “Popen”), cytoplasmic open (red lines, “Copen”) and occluded (magenta lines, “Occ”). DEER experiments were performed in the presence of the non-binding sugar NPGlc, which shifts the conformational equilibrium toward the cytoplasmic open state [11].

Having established that we have obtained a functionally, fully occluded state, we validated this model and the simulations by comparing them with distances derived from DEER experiments [11]. These have been previously interpreted as reporting the presence of cytoplasmic and periplasmic open states (revealed after the addition of the binding sugar, NPGal) and occluded conformations [27]. To better interpret these data, we have developed an approach (see Materials and Methods) that takes into account the flexibility of the spin label and thus predicts the electron spin–spin distance distribution that would be observed for a single structure or an entire MD trajectory. This is important as it allows us to compare like with like. We compared nine experimental distances to our simulations: three on the periplasmic side of the protein (residue pairs 105–310, 164–310 and 164–375; Fig. 5a) and six on the cytoplasmic side (residue pairs 73–401, 73–340, 136–340, 137–340 136–401 and 137–401; Fig. 5b). As the periplasmic open homology model [17] lacks some C-terminal residues, spin–spin distances 73–401, 136–401 and 137–401 can only be compared for the simulations of cytoplasmic open LacY.

The distance distributions calculated from the simulations of cytoplasmic open LacY agree well with the DEER experiments on LacY in the presence of NPGlc. This non-binding sugar ensures that the protein is found predominately in cytoplasmic open conformations [11]. In general, there is substantial overlap between the distributions calculated from simulations and the positions of the major peaks from the DEER experiments (Fig. 5, red lines *versus* gray histograms; Fig. S7a in the supplementary content). The absolute heights and widths of the experimental peaks are not expected to quantitatively agree with the simulations because the three independent simulations of individual conformational states do not capture the relative distribution of different states observed in a single experiment.

The distance distributions estimated from the simulation of periplasmic open LacY (blue lines in Fig. 5c) show the pattern expected for a periplasmic open state, that is, the spin label distances on the periplasmic side are shifted to larger values and the distances on the cytosolic side are shifted to smaller values compared to the equivalent distances estimated from the simulations of the cytoplasmic open transporter. One exception is distance 164–375 where the end of helix 5 with residue 164 moved closer to residue 375 on helix 11 at the beginning of the simulation and thus inverted the expected pattern. The distributions from the simulations generally overlap with the positions of the major experimental peaks for predominately periplasmic open LacY (Fig. 5c, blue lines; Fig. S7b).

Finally, we can validate the model of the occluded state against the DEER data. The spin–spin distance distributions calculated from the simulation *Occ* of the occluded model (Fig. 5c, magenta lines) are consistent with the experimental data. On the cytoplasmic side of the protein, the predicted spin–spin distances were shorter than those for cytoplasmic open LacY. The distributions for 73–340 and 136–340 coincided with minor peaks in the DEER experiments with NPGlc. These peaks are indicative of cytoplasmic closure as they overlap with the spin–spin distance distribution from the simulation of periplasmic open LacY where the cytoplasmic gate is closed. Peaks for 73–340, 137–340 and 136–340 for *Occ* also overlap with peaks from experiments recorded in the presence of NPGal that coincide with peaks for the simulation of the periplasmic open transporter (Fig. S7b in the supplementary content).

On the periplasmic side, the distances closely matched the distances for cytoplasmic open LacY (Fig. 5c, red lines), which is not surprising given that the periplasmic gate is closed. The peak for 164–310 for *Occ* closely follows the distribution from the simulations of cytoplasmic open LacY where the periplasmic gate is closed. For residues 164–310, the experimental DEER peaks at 31 Å and 37 Å in presence of binding and non-binding sugar have been attributed to conformations with both gates closed [27]. Encouragingly, the two peaks intersect the spin–spin distribution calculated from *Occ*.

### Order parameters allow comparison with crystal structures of MFS proteins

In order to compare our model of fully occluded LacY to known occluded MFS transporter structures such as PepT_So_ [32], XylE [30], [31], EmrD [28], NarU [29] and PiPT [33], we propose a pair of geometric order parameters. Putative gates were identified by comparing the experimental structures of cytoplasmic open LacY and the models of periplasmic open LacY [17]. The key feature of the closed periplasmic gate is the tight packing of helices 1 and 7, which seals off the central cavity to the periplasm. A similarly close packing of helices 4 and 10 is the crucial element of the cytoplasmic gate. Although other helices [32] have also been implicated in the gating of MFS transporters, we have focused on helices 1 and 7 and on helices 4 and 10 to capture the essence of the mechanism. The order parameter for the periplasmic gate is defined as the distance between the pair of C^α^ atoms in helices 1 and 7 with the shortest inter-helical distance in the wild-type crystal structure of LacY: this is I32–N245. The order parameter for the cytoplasmic gate is defined in a similar way, except that we now determine the pair of residues on helices 4 and 10 that shows the smallest C^α^–C^α^ distance in the homology model of periplasmic open LacY. The cytoplasmic order parameter is therefore defined as the distance between the C^α^–C^α^ atoms of residues E126 and C333. This choice of order parameters is consistent with previous studies of gating (see the supplementary content).

When the periplasmic and cytoplasmic gates are closed, the transmembrane helices 1 and 7 as well as helices 4 and 10 are kinked, enabling them to pack tightly against each other, sealing off any pathways across the membrane. Sequence motifs that facilitate helix bending by disrupting the transmembrane helices (such as prolines) are conserved across the MFS family [17], [63]. This observation suggests that key features of the gating mechanism could be shared between MFS transporters. By comparing the conformations of the gates in both the available crystal structures and our simulations, we tested the hypothesis that the overall conformational changes in the transport cycle are conserved across the MFS superfamily [17] (Table 2).

**Table 2:** Order parameters for MFS transporters.

Transporter	Cytoplasmic gate distance (C^α^–C^α^) TM4–TM10	Periplasmic gate distance (C^α^–C^α^) TM1–TM7
EmrD	Thr119 and Phe311	Ala32 and Cys229
FucP	Thr143 and Ser370	Gln51 and Ile282
GlpT	Trp138 and Ile368	Pro53 and Thr278
LacY	Glu126 and Cys333	Ile32 and Asn245
NarU	Met149 and Phe367	Ser54 and Ala273
NarK	Met151 and Phe370	Ser56 and Ala275
PepT_So_	Ser131 and Leu427	Thr36 and Ser320
PepT_St_	Ser130 and Gly407	Ala34 and Ser303
PiPT	Ala154 and Ala439	Asn53 and Leu332
POT	Asn138 and Gly420	Val47 and Thr314
XylE	Met149 and Ser396	Ser32 and Tyr298
YajR	Ser125–Ser328	Thr37 and Pro236

**Fig. 6:**
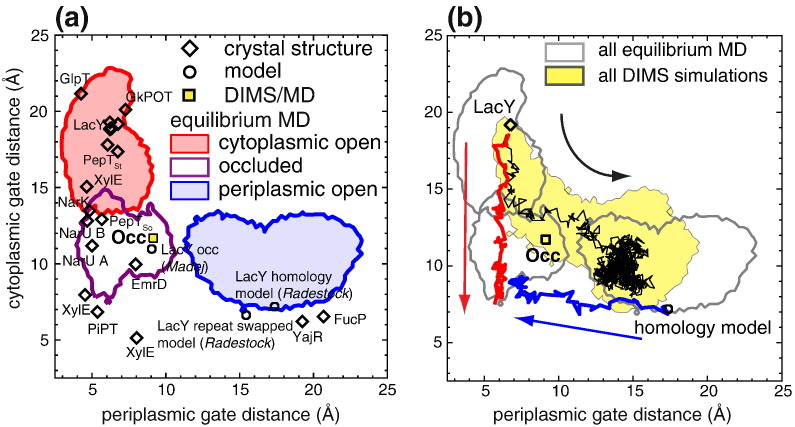
Order parameters. MFS transporter structures can be characterized by two order parameters: the cytoplasmic and periplasmic gate openings (see the text). (a) Individual structures are shown together with equilibrium MD simulations: experimental crystal structures (diamonds with labels), models of LacY (circles with labels) [17], [27] and our occluded model generated by DIMS and equilibrium MD (square). The red area indicates the range of order parameter values sampled in the simulations started from the cytoplasmic open state (LacY crystal structure 2V8N [14]). Similarly, the purple outline corresponds to simulation *Occ* started from the occluded DIMS structure and the blue area shows the range of all simulations started from the FucP-based homology model by Radestock and Forrest [17] in the periplasmic open state. (b) The range of order parameters covered by an ensemble of DIMS MD simulations from the cytoplasmic open state (LacY) toward the periplasmic open state (homology model) is shown as a yellow-filled area (simulations *DIMS-c.open-p.open*). A typical DIMS transition is drawn as a black thin line. Occluded conformations were generated by special DIMS simulation that either started from the cytoplasmic open state and closed the periplasmic gate (red line, simulation *DIMS-closure*) or began from the periplasmic open state and closed the cytoplasmic gate (blue line). The endpoint of *DIMS-closure* was simulated for 100 ns with equilibrium MD [simulation *Occ*; purple area in (a)] and resulted in the structural model for the occluded state labeled “Occ” (square). The ranges of order parameters sampled by the equilibrium simulations in (a) are shown as gray outlines.

The order parameters not only tracked the state of the gates in the simulations but also consistently ordered all the available MFS transporter structures (Fig. 6a). For cytoplasmic and periplasmic open LacY, the order parameters agreed with both the pore profile and the water density analysis. The equilibrium simulation of the occluded structure, *Occ*, sampled a region of order parameter space (Fig. 6a) intermediate between cytoplasmic and periplasmic open LacY. The cytoplasmic order parameter remained predominately < 13 Å while the periplasmic order parameter was < 8 Å. Both gates were shut, which is consistent with the calculations of the pore radius (Fig. 3b).

The order parameters intuitively classify all the available MFS transporter crystal structures (Fig. 6a) even though the pairwise sequence identify between members of the MFS is typically low, for example, no more than 12% between LacY and any of the other transporters studied here [64] up to a maximum of 46% for the closely related peptide transporters PepT_St_ and GkPOT [65]. The cytoplasmic open crystal structures for LacY, GlpT, PepT_St_ and GkPOT and the inward open structure of XylE mapped onto the region of order parameter space sampled by the simulations of cytoplasmic open LacY. The two virtually identical FucP crystal structures were close to the region of order parameter space explored by the simulations of periplasmic open LacY, while the recently solved periplasmic open structure of YajR was located slightly farther outside. The crystal structures of NarK and PepT_So_ border on the region of order parameter space sampled by the simulations of cytoplasmic open LacY during which it partially closed, while NarU B is only slightly removed, all of which is consistent with the partially cytoplasmic closed structure of LacY constituting a transition intermediate [25]. Importantly, the occluded crystal structures of PepT_So_, EmrD, NarU (A and B) and NarK and cytoplasmic partially occluded structure of XylE mapped to the intermediate region accessed by *Occ*, with the occluded structure of PiPT close by. Classifying the crystal structure of periplasmic partially occluded XylE [30] using our order parameters puts it outside the region sampled by *Occ*. While the periplasmic order parameter for XylE was similar to EmrD (~ 8.0 Å), the cytoplasmic order parameter for XylE was very low, ~ 5.1 Å. This is much lower than the cytoplasmic order parameters seen in our simulations or any other MFS transporter structure and probably reflects the presence of the additional intracellular helices that pack close to the cytoplasmic gate and that are not present in the other MFS transporter structures.

In order to assess the robustness of our conclusions, we introduced a fairly drastic change into our equilibrium simulations by changing the underlying force field from GROMOS96 to CHARMM22. As discussed in the supplementary content, both sets of simulations behave very similarly and, in particular, cover similar regions of order parameter space (Fig. S8b). Thus, the order parameter analysis and the simulations are robust against changes in the simulation parameters and are therefore likely to reflect upon a real property of the LacY protein, namely, the flexibility of its cytosolic and periplasmic gates.

Taken together, our analysis suggests that key features of the gating mechanism are conserved across the MFS transporters and a meaningful structural comparison is possible even in cases such as XylE where additional structural elements interact with the gates. In particular, our model for the occluded conformation of LacY is consistent with the known structural evidence for occluded conformations in the MFS superfamily, as revealed by our order parameter analysis.

In a recent study, Madej et al. modeled the occluded state of LacY by homology to the crystal structure of partially occluded PepT_So_ [27]. Even though their model and ours were derived by completely different methods, they are generally quite similar with a C^α^ RMSD of 4.4 Å computed with PyMOL (Schrödinger, LLC); the agreement is higher for the C-terminal domain (3.2 Å) than for the N-terminal domain (4.8 Å). The similarity is also emphasized by the order parameters for the gates of the occluded homology model. The cytoplasmic and the periplasmic gates are closed in the homology model and the model maps onto the region of the two-dimensional order parameter plot (Fig. 6a) populated by occluded structures from our simulations, both from the equilibrium simulation of the occluded state, *Occ*, and from the simulation of the transition between cytoplasmic and periplasmic open LacY (*DIMS-c.open-p.open*) described in the next section.

### DIMS transitions between cytoplasmic and periplasmic open LacY

In order to test the hypothesis that the conformational change proceeds via an intermediate occluded state, we generated an ensemble of 80 transitions between the end states. These DIMS MD simulations (*DIMS-c.open-p.open*) started with the cytoplasmic open wild-type crystal structure of LacY and used as the target structure the entire N- and C-terminal domains (residues 7–185 and 220–399) of the homology model of periplasmic open LacY. During the DIMS MD simulations, the structure of the transporter changed from a cytoplasmic open/periplasmic closed state to a cytoplasmic closed/periplasmic open conformation as shown by analysis of pore radius profiles (Fig. S11 in the supplementary content). These transitions connect the broad wells sampled by the equilibrium MD simulations of cytoplasmic and periplasmic open LacY in our order parameter space (Fig. 6b). Intermediate structures in the DIMS transitions between cytoplasmic and periplasmic open LacY were occluded according to both analysis of the pore radius (Fig. S11) and the order parameters (Fig. 6b). These structures occupy a region in order parameter space that is sampled by the *Occ* equilibrium MD simulation (Fig. 6a and b). Thus, two different DIMS MD simulations produced structures with both their gates impermeable to H^+^ and sugar: a full transition between cytoplasmic and periplasmic open LacY (*DIMS-c.open-p.open*) and a transition using partial structural information to close the cytoplasmic half of cytoplasmic open LacY (*DIMS-closure*) together with subsequent equilibrium MD.

### Coupling of the flexible gates

The results of our simulations lie somewhere between a strict rocker-switch mechanism, where the gates open simultaneously [1], [16], and a strict airlock mechanism [20], where the gates open simultaneously (Fig. 1b). We would expect a single, diagonal straight line to connect the cytoplasmic and periplasmic open states in the order parameter plot (Fig. 6) in the case of the former and an “L” shape for the latter. The simulated transitions between the cytoplasmic open and periplasmic open conformations using DIMS (*DIMS-c.open-p.open*) did not fall on either set of hypothetical lines. We were able, however, to close one gate independently of the other by only applying a bias to the open gate (*DIMS-closure*; Fig. 6b), which highlights the structural flexibility in LacY and is consistent with a gating motion more similar to an airlock mechanism. A simple structural comparison also argues against the rigid-body motion implied by a literal rocker-switch mechanism. Significant intra-domain arrangements (all with C^α^ RMSD > 2.9 Å) are apparent when comparing the N- and C-terminal domains of representative structures for the three key states—cytoplasmic open, periplasmic open and occluded (Fig. S12).

The conformational flexibility detected by previous studies also argues against a literal rocker-switch mechanism and is consistent with the results from our simulations. The conformational flexibility of LacY has been previously highlighted in hydrogen/deuterium exchange experiments [21], [22], [23] and in intra-domain cross-linking experiments [66], [67], which argue for high intra-domain flexibility. Furthermore, equilibrium MD simulations of LacY have suggested that its individual helices are flexible [18], [25] during the initial stages of the conformational switch. SGLD simulations [44] of LacY have shown intra-domain re-arrangements of the helices, as well as global motions during the transition itself, consistent with our conclusions.

In summary, our simulations provide multiple lines of evidence for the existence of a functionally occluded state in LacY, as hinted at by a range of experimental studies. A model for the intermediate occluded state was derived using molecular simulation that only utilized the crystal structure of the cytoplasmic open conformation and a homology model of the periplasmic conformation. It agrees with the available structural information on occluded states of MFS transporters and experimental DEER data. Residues known to be involved in the transport cycle occupy positions predicted in other studies. Calculation of geometric order parameters enabled comparison of all MFS transporter X-ray crystal structures, an approach that will become increasingly fruitful as more structures become available. The movements of flexible gates were seen to be coupled and thus lead to the formation of occluded transition intermediates. While global motions of the N- and C-terminal domains relative to one another do play a role, we rule out a strict “rocker-switch” mechanism involving rigid domain movements. We have shown that it is productive to describe LacY (and by extension, the other MFS transporters) as multi-gated pores [68] by (1) assessing the barriers to substrate permeation for different states in the transport cycle and (2) quantifying the changes in the gates by order parameters. A similar approach has already proven successful for the LeuT-like transporters [58], [69], [70] and could therefore act as a general paradigm to computationally analyze secondary transporter function.

## Materials and Methods

### Membrane protein insertion

The LacY wild-type crystal structure (PDB code 2V8N [14]) was downloaded from the Protein Data Bank. The homology model of Radestock et al. [17] for periplasmic open LacY (PM0077183), which is based on the periplasmic open structure of FucP, was obtained from the protein model database [17], [71]. These structures, as well as a model for a fully occluded structure of LacY obtained from DIMS simulations, were inserted into POPC bilayers by an established protocol [51], [72], [73]. In all structures, His322 was neutral as suggested by the proposed mechanism [2]and modeled as the imidazole N_ε_ tautomer except for simulations *COpenB1* and its continuation *COpenB2* in which the N_δ_ tautomer was selected by the pdb2gmx tool [74] as more favorable. A distance cutoff of either 7 Å or 10 Å was used for the elastic network model that preserves protein structure in the coarse-grained insertion protocol. Self-assembly simulations were run for 200 ns. The coarse-grained systems were then converted into atomistic systems, by superimposing the original atomistic structures onto the coarse-grained protein structure. The overall system charge was neutralized and additional Na^+^ and Cl^−^ ions were added, resulting in a physiological free NaCl concentration of 100 mM.

### Atomistic equilibrium simulations

Atomistic simulations were run in GROMACS 4.5 [74], either with the GROMOS96 43a1 [75] force field with Berger's lipid parameters [76] (“GROMOS”) or with the CHARMM22 protein force field [77], including the CMAP correction [77], [78] and CHARMM36 lipid parameters [79] (“CHARMM”). All atomistic simulations were performed at 315 K and 1 bar. The Parrinello–Rahman barostat was used for semi-isotropic pressure coupling [80]. Temperature coupling was achieved by velocity rescaling with a time constant of 0.1 ps [81]. The P-LINCS algorithm was used to constrain bonds involving hydrogen atoms [82]. The SPC [53] and the CHARMM TIP3P [83] water models were used for atomistic simulations with GROMOS and CHARMM, respectively. The radius for neighbor list searches was set to 10 Å and 12 Å, respectively, for simulations with GROMOS and CHARMM force fields. Long-range electrostatics were simulated with the particle mesh Ewald method [84], either with a real-space cutoff of 10 Å (GROMOS) or a switching function between 8 Å and 10 Å (CHARMM). Van der Waals forces were cut off at 10 Å (GROMOS) or switched off between 8 Å and 10 Å (CHARMM). Production runs were preceded by energy minimization and 5 ns equilibration using position restraints with a harmonic force constant of 1000 kJ/mol nm^2^ on the protein heavy atoms.

### Cluster analysis

The simulation of the occluded conformer of LacY was analyzed using the algorithm by Daura et al. [85] in order to pick relevant frames for further analysis. All protein atoms were considered in the geometric clustering and a distance cutoff values of 0.25 nm was employed. The structure used for electrostatics calculations—the final structure of the trajectory *Occ*—was part of the dominant cluster with a cutoff of 0.25 nm.

### DIMS MD

DIMS MD [40], [41] was used to sample transitions between cytoplasmic and periplasmic open LacY. Simulations were run with the CHARMM 36a1 program using the CHARMM22 all-atom force field [77], [86]. A soft ratcheting bias Δφ = 10^− 4^ Å was used in *DIMS-c.open-p.open* and Δφ = 10^− 6^ Å was used in *DIMS-closure*. Langevin dynamics were run at 300 K with a timestep of 2 fs and a friction coefficient of 25.0 ps^− 1^. For efficiency, we modeled the protein environment with the GBSW implicit solvation model [46], [47], which has previously been used to obtain DIMS transitions for the KcsA ion channel in a membrane environment [57]. The CMAP dihedral correction and atomic radii optimized for GBSW were used [87]. The GBSW model approximates the bilayer as an exclusion zone from the aqueous phase (ε = 80) [46]; here, we employed a GBSW membrane thickness of *h*_memb_ = 40 Å and a smoothing length of *w*_m_ = 6.5 Å (Fig. 2c) to approximate the POPC membrane geometry seen in the explicit solvent simulations (Fig. 2d). The dielectric changed smoothly from ε = 1 to ε = 80 in the head group region. The protein was positioned in the implicit membrane by structural alignment to a fully atomistic system generated by the membrane insertion protocol described above using MDAnalysis [90], [91], [92] and energy minimized. The half-smoothing length *sw* for the dielectric boundary was set to the default of 0.3 Å and the non-polar solvation contribution *s*γ was 0.04 kcal/(mol Å^2^). The default non-bonded options for GBSW simulations were employed, with an inner cutoff of 16 Å and a cutoff for the energy calculation of 16 Å. A cutoff for non-bonded list generation of 20 Å was used. The RMSD of the non-hydrogen backbone atoms for the N-terminal domain (residues 7–185) and the C-terminal domain (residues 220–399) to the target conformation was used as a progress variable in DIMS. The target structure was reoriented relative to the current structure every 10 MD steps. Non-hydrogen backbone atoms of the N- and C-terminal domains were used for rotational and translational fit. To close just the cytoplasmic or periplasmic gates, we used only atoms with *z* < 0 Å or *z* > 0 Å (where *z* = 0 Å marks the midplane of the membrane) for the RMSD calculation assessing the change in progress variable that drives the soft ratcheting bias. Eighty simulations were performed with differing seeds for the random number generator that drives the stochastic term of the Langevin equation so that each simulation sampled a different microscopic pathway.

### HOLE analysis

Centered and fitted trajectories were used for pore radii analysis of equilibrium MD trajectories. Conformations were written out every 0.1 ns and their radial pore profiles were calculated with the program HOLE [50]. NumPy [88] was used to histogram the pore profiles, which allowed the calculation of average pore profiles and standard deviations, and figures were plotted with Matplotlib [89]. All frames from the 80 DIMS trajectories between cytoplasmic and periplasmic open LacY were analyzed by HOLE. For each structure, the backbone RMSD distance of the N-terminal domain (residues 7–185) and the C-terminal domain (residues 220–399) to the periplasmic open target was calculated with MDAnalysis [90], [91], [92]. The HOLE profiles and the RMSD distances were gridded and interpolated with Matplotlib to be shown as contour plots. Python code for the pore radius analysis is available as part of the HOLE analysis module of the MDAnalysis library [90].

### Water density analysis

Water densities were calculated for centered and RMS-fitted trajectories using the program gridcount^1^ [52]. The densities were represented using the volume viewer in UCSF Chimera [54], [55] and measured relative to the density of SPC bulk water at standard conditions, 0.970 g/cm^3^ [93].

### DEER distance analysis

DEER is an EPR technique for measuring distances between two spin labels that have been covalently attached to a protein. Two cysteine residues are introduced into the protein and subsequently labeled. The positions are chosen to report on the expected conformational change. The study by Smirnova et al. used (1-oxyl-2,2,5,5-tetramethylpyrroline-3-methyl)-methanethiosulfonate (MTSL) spin labels [11]. MTSL has a linker with five rotatable bonds and is therefore very flexible. The distance distributions between the two spin labels measured by the experiment for LacY are typically broad and often multi-modal. The distributions are therefore a convolution of the flexibility of the MTSL spin label and the conformational spread of the proteins in the sample. To ensure that we compared like with like, we developed a method that (1) maps rotamer libraries of the MTSL spin label onto each position, (2) discards those rotamers that sterically clash with the protein (typically distances < 2 Å) and (3) calculates all (weighted) distance pairs between the remaining rotamers and (4) thereby estimates a distance distribution for that structure. The code was written in Python using the MDAnalysis library [90] and incorporates a published rotamer library for MTSL [94]. It is available for download from the MDAnalysis website^2^. Our approach improves upon an existing method [94] by increasing computational efficiency and implementing, via the MDAnalysis library [90], analysis of ensembles of hundreds of structures, which allowed us to estimate distance distributions for entire simulation trajectories. For spin–spin distance pairs 73–340, 136–340 and 137–340, a clash distance cutoff of 1.5 Å was used. The estimated distance–distance distributions were then compared to the previously reported DEER measurements of LacY [11].

### Order parameter analysis

Two order parameters that report on whether a MFS transporter structure is cytoplasmic or periplasmic open were defined for EmrD (PDB code 2GFP) [28], FucP (3O7Q, 3O7P) [16], GlpT (1PW4) [95], cytoplasmic open LacY (1PV6, 1PV7, 2CFQ, 2CFP, 2V8N, 2Y5Y) [12], [13], [14], [15] or periplasmic open LacY (homology model: PM0077183, repeat swapped model: PM0076824) [17]. NarU (4IU8) [29], NarK (4JR9) [96], PepT_So_ (2XUT) [32], PepT_St_ (4APS) [97], PiPT (4J05) [33], POT (4IKV) [65], XylE (4GBY, 4JA3, 4JA4) [30], [31] and YajR (3WDO) [98] are summarized in Table 2. Distance matrices were calculated for C^α^ atoms of the pairs of transmembrane helices 1 and 7 for the closed periplasmic gates of LacY, GlpT, EmrD, NarK, NarU, PepT_So_, PiPT, POT and XylE using MDAnalysis [90] (see Table S2 for definitions of the transmembrane helices). The closest contact defined the C^α^–C^α^ distance that serves as the order parameter. Similarly, the cytoplasmic order parameter was calculated for the closed cytoplasmic gates of FucP, EmrD, NarU, PepT_So_, PiPT, XylE and YajR, as well as the homology and the repeat swapped model for periplasmic open LacY. No structure featuring the closed cytoplasmic exists for GlpT, NarK, PepT_St_ and POT. Similarly, the structures of the closed periplasmic gates of FucP and YajR are unknown. The order parameters were defined via structural alignments. GlpT, NarK, PepT_St_ and POT were aligned with cytosolic open LacY by the DALI pairwise alignment server [99], which was also used to align YajR to the periplasmic open homology model of LacY. For FucP, the refined structural alignment with cytoplasmic open LacY obtained by Radestock et al. [17] that underlies the homology model for periplasmic open LacY was used.

### Poisson-Boltzmann calculations

In order to assess the energetics of proton permeation through aqueous pathways in a semiquantitative manner, we computed the position-resolved electrostatic free energy *W*_elec_(*r*) for a solvated proton on a 3D grid of ion positions *r*. Proton transport in aqueous solution involves a complicated “fluxional complex” in which the Eigen (H_9_O_4_^+^) and Zundel cation (H_5_O_2_^+^) hydration structures of the proton rapidly interconvert [100]. In order to quantify the energetics of solvated protons inside a membrane protein, we adopted the much simpler Born description, which treats the proton as a cavity in a continuum solvent with an effective radius *a* and a charge of + 1. The use of such a simple model is motivated by the insight that the energetics are likely dominated by the electrostatics of the charge in the low dielectric membrane compared to smaller differences due to the detailed proton–water interactions. The effective Born radius of the solvated proton was estimated as *a* = 1.4848 Å by solving the Born equation *a* = –166 Å kcal mol^–1^ *q*^2^/ΔG*_solv_ (1–1/80) with the experimental hydration free energy of the oxonium ion (H_3_O^+^) at the 1 M standard state, ΔG*_solv_ = –110.4 ± 0.7 kcal/mol [101].

Within the framework of Poisson-Boltzmann theory, *W*_elec_(*r*) is the electrostatic free energy of the ion bound to the protein compared to the ion and protein in bulk solution separately:

$$\Delta W_{\mathrm{elec}}\left(r\right)=G_{\mathrm{protein}+\mathrm{ion}}\left(r\right)-G_{\mathrm{protein}}-G_{\mathrm{ion}}$$

In the case of membrane proteins, the reference energy *G*_protein_ is calculated for the protein embedded in a membrane-like environment as described below. Such an approach has been used previously to calculate the electrostatic free-energy profile (“Born profile”) for ions in channels and pores along the permeation pathway [45]. Here this concept is extended to generate the 3D free-energy landscape of an ion in the heterogeneous environment of the LacY transporter. The linearized Poisson-Boltzmann equation was solved with APBS [102] at an ionic strength of 100 mM NaCl and a temperature of 298.15 K for ion positions *r* generated with a customized version of HOLLOW [103] at a point spacing of 1 Å. A dielectric constant of ε = 80 was assigned to bulk water and a dielectric constant of ε = 10 was assigned to the protein. An implicit membrane was modeled after the membrane observed in the MD simulations as a low dielectric slab with a hydrophobic core of thickness 33.5 Å with ε = 2 and a head group region of thickness 6.5 Å with ε = 20 (Fig. 2c). The water-accessible interior of the transporter was modeled as a high dielectric (ε = 80). Manipulation of the dielectric maps was carried out with software based on the *draw_membrane* tool in APBSmem [104] and an updated version of the *BornProfiler* analysis package [45]. The calculations employed a manual focusing strategy in three steps on a computational grid of 129 × 129 × 129, starting from a cubic bounding box of length 250 Å, via a 100-Å box, to a final dimension of 50 Å, centered on the position of the ion. Increasing the computational grid to 161 × 161 × 161 did not change the energies and hence the results can be considered converged with respect to the grid. Starting structures for simulations *COpenA1* and *POpenB*—which had been equilibrated in atomistic membranes and are fully cytoplasmic and periplasmic open respectively—as well as final structures from MD simulations of *COpenA1*, which showed partial closure, and fully closed *Occ* were used for Poisson-Boltzmann calculations. Partial charges and radii from the CHARMM force field were assigned with pdb2pqr [105]. All ionizable residues were simulated in their default charge state as either suggested by PROPKA [106] or because the default protonation state better described a transporter in that part of the transport cycle that is believed to occur without involvement of substrate or proton transport [2].

### Accession number

A representative structure of the occluded conformation (the last frame of the simulation *Occ*) has been deposited in the protein model database [71] under accession code PM0079313 and is also available in the supplementary content.

## Appendix A Supplementary Data

Supplementary material 1 Model of the occluded conformation of apo-LacY in PDB format.

## Footnotes

1 available from https://github.com/orbeckst/gridcount

2 http://mdanalysis.googlecode.com
