# Supplementary material for: Flexible Gates Generate Occluded Intermediates in the Transport Cycle of LacY
Source: J Mol Biol. 2014 Feb 6;426(3):735–51. doi: 10.1016/j.jmb.2013.10.024 (PMC3905165; doi:10.1016/j.jmb.2013.10.024)
Supplement: Supplementary file 2 — Supplementary material 2 [file mmc2.pdf]

Supplementary Information

Here we show further analysis and additional simulations (see Table 1 in the main text). All equilibrium simulations discussed in the main text used the GROMOS96 43a1 force field[4] (GROMOS) with the Berger lipid parameters[11]. To illustrate the robustness of our conclusions we show additional data from repeat simulations and from simulations of the cytoplasmic open and periplasmic open transporter with the CHARMM 22 force field with the CMAP correction[14] and the CHARMM 36 parameters for lipids[15]. In general, GROMOS and CHARMM simulations behave similarly in the quantities of interest analyzed in this work, as shown below

RMSD analysis of the MD simulations

Structural drift in the equilibrium MD simulations was assessed by calculating Cα RMSDs for the N- and C-terminal domains (residues 7-185 and 220-399), to the starting structures of simulations *COpenA1*, *Occ* and *POpenA*. Flexible regions such as the inter-domain linker were excluded from the analysis.

The Cα RMSDs calculated for the simulation *COpenA1* stabilised around 3 Å (Figure S1A), as the protein relaxed away from the crystal structure in an atomistic lipid membrane. The homology model for periplasmic open LacY was less stable with the RMSD rising to values ~5 Å (Figure S1B). Not all interactions stabilising periplasmic open LacY might have been captured in the homology model, which would not be surprising given the low sequence identity between LacY and FucP (~10%)[17; 18] For example, the side-chains of E325-R302 are thought to interact in the periplasmic open transporter but cannot interact in the current model. Nonetheless the model captures the overall features of the periplasmic open transporter, is reasonably stable (see also the DSSP analysis below) and compares well to the available experimental data[19] and is thus an excellent starting point for our study.

The RMSD calculations also confirm that our model for the occluded conformation is a distinct and stable conformational state. The Cα RMSD curve for *DIMS-closure* rises to ~4 Å (Figure S1C) which shows that structural changes that accompany the closure of the cytoplasmic gate go beyond the more limited changes (Cα RMSD stabilizing at ~3 Å for *COpenA1*) associated with the initial closure of LacY in the equilibrium simulations. As the occluded structure is relaxed into an atomistic bilayer in an equilibrium simulation (*Occ*), the Cα RMSD to the crystal structure stays close to ~4 Å (Figure S1D), which means that no further drift away from the cytoplasmic open structure is observed. The Cα RMSD to the final frame of the DIMS simulation of the closure of the cytoplasmic gate (*DIMS-closure*), levels off at ~3 Å (Figure S1D). The occluded structure derived from molecular simulations is comparable in stability to the crystal structure of cytoplasmic open LacY as judged by Cα RMSD.

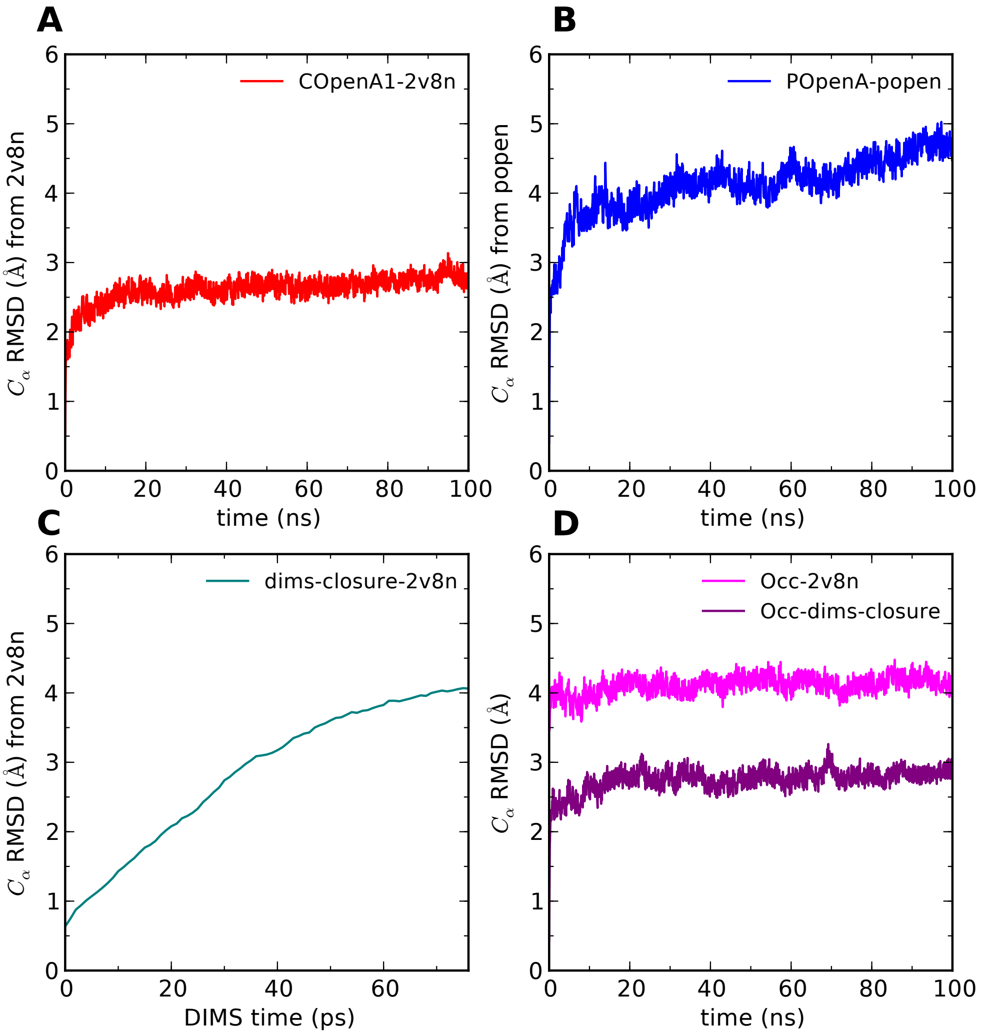

Figure S1: Cα RMSDs for representative simulations *COpenA1*, *Occ*, *POpenA* and *DIMS-closure*. The Cα RMSD was calculated for the structured N- (7-185) and C-terminal domains (220-399) of LacY omitting the flexible linker, using the MDAnalysis library. The simulations were compared to their respective starting structures, the 2V8N crystal structure for *COpenA1* (**A**) and *DIMS-closure* (**C**), the periplasmic open homology model based on FucP for *POpenA* (**B**). The simulation of the occluded structure, *Occ*, was compared to its starting conformation (**D**), the cytoplasmic and periplasmic closed final frame of *DIMS-closure* (in purple).To emphasize that *Occ* is in an sense a continuation of *DIMS-closure*, we also show the *Occ* trajectory's Cα RMSD relative to the 2V8N crystal structure in **D**

Analysis of secondary structure stability

The stability of the simulations was also assessed by analysing changes in secondary structure over the course of the simulations. The secondary structure (assigned by DSSP[9]) shows some local breaking of α-helices (Figure S2) but overall the protein retains its transmembrane helices. In particular, the simulation of the putative occluded state, *Occ*, maintains a very well defined secondary structure (Figure S2C), with some helices such as TM VIII being even more helical than in the simulation based on the periplasmic open model (Figure S2B). The CHARMM simulations (Figure S2 D, E) appear to maintain secondary structure to a slightly higher degree than the GROMOS simulations but show the same picture overall.

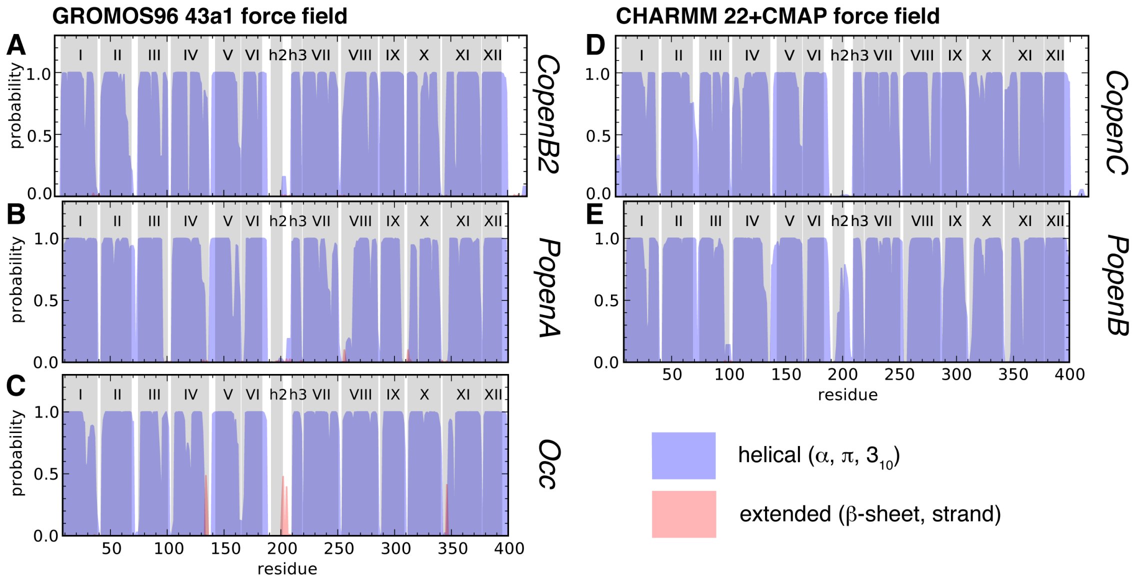

Figure S2: Secondary structure in equilibrium MD simulations. Secondary structure elements assigned by Abramson et al[2] are labelled and shown as gray bars (transmembrane helices I–XII and solvent-exposed helices h1–h3). The probability to observe a particular secondary structure for a given residue (as defined by DSSP[9]) over a equilibrium MD trajectory is shown as blue (any helix) or red (strand/sheet) filled curves. (Figures generated with *GromacsWrapper* (Oliver Beckstein, https://github.com/orbeckst/GromacsWrapper) from data obtained by the Gromacs tool do_dssp[13]). Selection of simulations that used the GROMOS96 43a1 force field: (**A**) Simulation *CopenB2* of cytoplasmic open LacY. Data are representative for *COpenA1, COpenA2,* and *COpenB1* (data not shown). (**B**) Simulation *PopenA* of periplasmic open LacY. (**C**) Simulation *Occ* of occluded LacY. Simulations that used the CHARMM 22 + CMAP force field: (**D**) Simulation *CopenC* of cytoplasmic open LacY. (**E**) Simulation *PopenB* of periplasmic open LacY. Note that secondary structure element h2 was defined for the first LacY structures (1PV6, 1PV7)[2] but the region appears unstructured in the higher resolution crystal structure 2V8N[16] that formed the basis for the simulations reported here

Additional HOLE profile and water density analysis

The partial closure of the cytoplasmic gate from a radius of 4 Å in the crystal structure to about 1 Å, which was observed in simulation *COpenA1* (Figure 3A in the main text) is maintained over another 100 ns (*COpenA2*, and extension of simulation *COpenA1*) as seen from the pore radius profile in Figure S3A. A repeat of the simulation (starting again from the crystal structure) but using a larger membrane patch (Figure S3B) and also when using the CHARMM force field (Figure S3C) confirmed the partial closure, although only to a minimum radius of ~2 Å. The simulation of the periplasmic open state with the CHARMM force field showed a similar partial closure of the periplasmic gate from 4 Å to slightly above 2 Å. Taken

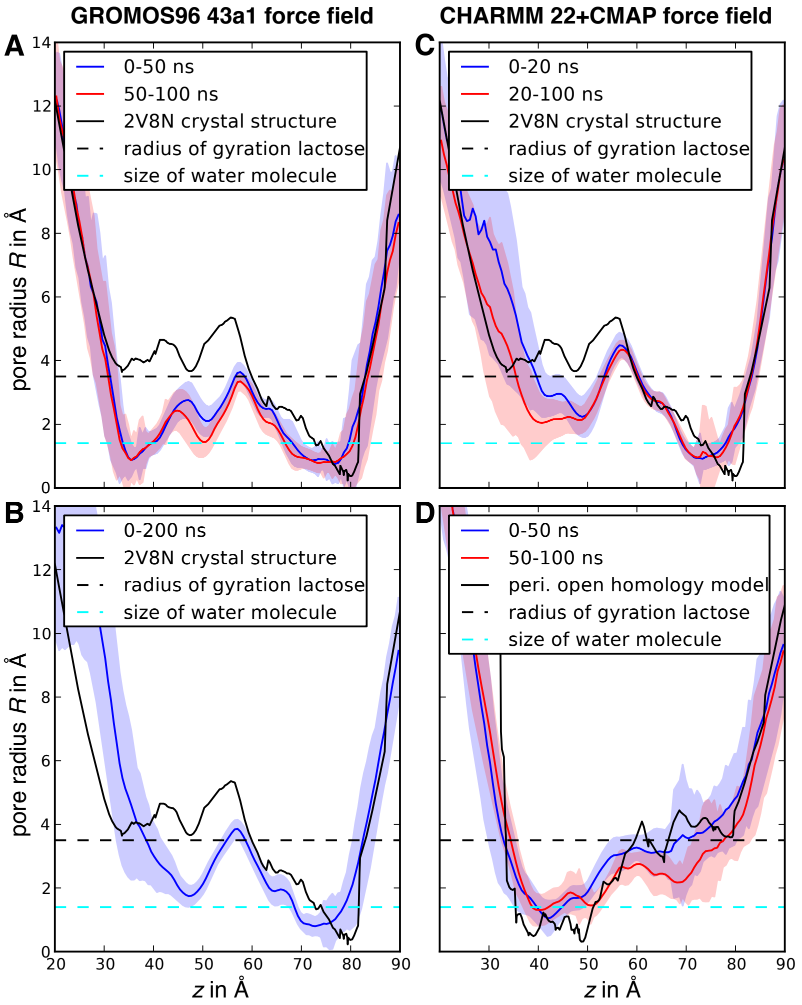

Figure S3: Pore profiles. Pore radii determined by HOLE[1] are plotted against *z*-coordinates of the cavity for each simulation and the crystal structure (2V8N) or the FucP-based homology model of the periplasmic open state. The average of the pore radius profile over the indicated time of the trajectory is plotted as a solid line together with the standard deviation as a shaded band. For scale, the size of a water molecule (cyan dashed line) and a lactose molecule (black dashed line) are indicated. (**A**) Simulation *COpenA2* (100-ns extension of *CopenA1*, shown in Figure 3A in the main text) (**B**) Simulation *COpenB2.* (**C**) *COpenC* (CHARMM force field, cytoplasmic open). (**D**) *POpenB* (CHARMM force field, periplasmic open).

together, these simulations all indicate that the gates of LacY are rather flexible and at least partially mobile on the 100-ns time scale.

Despite the reduction of the radius of the access pathway from 4 Å to about 2 Å as observed in the simulations *COpenB2*, *COpenC*, and *PopenB* (Figures S3C, B, D) there was still continuous water density visible, as shown by the water density analysis (Figures S4C, B, D). Thus, even in cases where partial gate closure was observed, a continuous water pathway existed from the bulk region to the central cavity. Therefore, neither the repeat simulations nor the ones using a different force field produce a true occluded state.

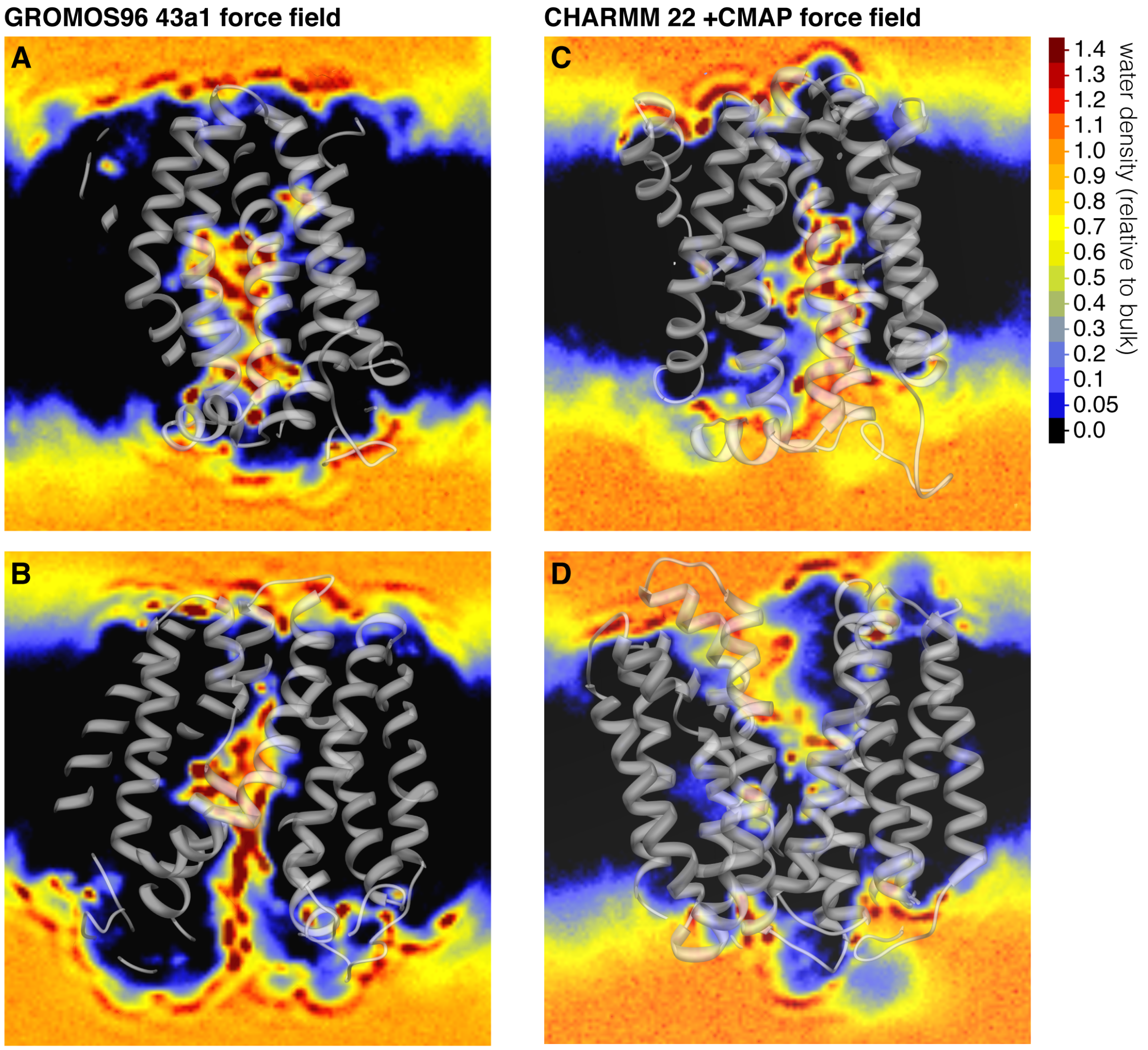

Figure S4: Water density. Slices through the water density (relative to bulk SPC water [3]) are shown together with the protein in gray cartoon representation. Selected simulations performed with the GROMOS96 43a1 force field (see also Figure 3D–F in the main text): (**A**) *COpenA2.* (**B**) *COpenB2.* Simulations performed with the CHARMM 22 + CMAP force field: (**C**). *COpenC.* (**D**) *POpenB.* Visualised in USCF Chimera[7; 8].

Evaluation of the occluded model from DIMS and MD simulation

The structural quality of our model for the occluded conformer of LacY was evaluated using the Molprobity server [20]. The quality of the model is comparable to the 3.6 Å crystal structure (2V8N) of cytoplasmic open wild-type LacY [16] (See Table S1). It performs better than the 2V8N crystal structure in respect to rotameric states and Ramachandran plot statistics, while Cβ and backbone geometry is worse than in the wild-type crystal structure although it needs to be remembered that this model is a snapshot from an equilibrium MD simulation at finite temperature and hence bonds and angles are expected to slightly diverge from ideal geometry due to thermal fluctuations.

Table S1: Molprobity quality evaluation of the 2V8N crystal structure and the model for occluded LacY

	Cyt. open LacY (2V8N, chain A, residues 1-417)	Model for occluded LacY (DIMS+MD, residues 7-399)	Goal
Poor rotamers	53 (15.32%)	25 (8.56%)	< 1%
Ramachandran outliers	74 (17.83%)	16 (4.09%)	< 0.05%
Ramachandran favoured	228 (54.94%)	314 (80.31%)	> 98%
Cβ deviation >0.25 Å	0 (0%)	62 (17.27%)	0
Bad backbone bonds	0/1667 (0%)	5/1570 (0.32%)	0%
Bad backbone angles	8/2082 (0.38%)	41/1961 (2.09%)	<0.1%

Electrostatic free energy of the partially occluded conformation

The electrostatic free energy landscape of a solvated proton in cytoplasmic open LacY as represented by the crystal structure (PDB id 2V8N) was shown in the main text in Figure 3G. However, all our simulations show a partial closure of the cytoplasmic gate and even though the water density analysis indicates the presence of an aqueous pathway (Figures 3D and S4A–C) it was not obvious if this was sufficient for a solvated proton to permeate into the central cavity. Therefore, we performed the same Poisson-Boltzmann analysis for the most constricted of the partially occluded

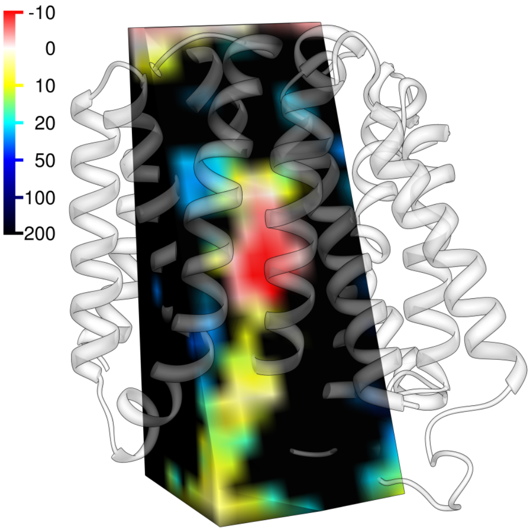

Figure S5: Electrostatic free energy for H^+ permeation through cytoplasmic open LacY after the initial closure. The position-resolved electrostatic free energy $W_{elec}(r)$ for solvated H^+ interacting with the final structure of simulation *COpenA1* calculated with BornBrofiler[5] and visualised in USCF Chimera[7; 8] is shown. Energies are in kJ/mol.

conformations, the last frame (at 100 ns) of simulation *COpenA1*. As Figure S5 shows, the electrostatic barriers along the whole pathway remain below 10 kJ/mol. The cavity itself remains at least as attractive (~ –10 kJ/mol) for the solvated proton as seen for the crystal structure (Figure 3G).

Thus, even though the geometry of the pathway suggests occlusion (radius < 1 Å), analysis of the water density (Figure 3D) and the electrostatic calculations shown here (Figure S5) indicate that at least protons could still enter or exit from the central binding site. Hence the partially occluded conformation obtained from the equilibrium simulations should not be considered a functionally occluded state.

The E325-R302 salt-bridge in the simulations

The establishment of a salt-bridge between the E325 and R302 is thought to facilitate the switch from cytoplasmic to periplasmic open LacY. This salt-bridge is present neither in the cytoplasmic open crystal structure nor the periplasmic open homology model that were the inputs for our simulations. Plotting the minimum distance between nitrogen atoms of the guanidinium group of R302 and the oxygen atoms of the E325 side-chain, for simulations *COpenA1* and *POpenA*, shows that no stable salt-bridge was formed (Figure S6A) during the course of the simulations. The average separation remained of ~5.1±0.6 Å in *COpenA1*. E325-R302 remained at a large separation of ~14.0±0.9 A in *POpenA.* Closing the cytoplasmic gate in a DIMS simulation (DIMS-closure) brought the two side-chains closer together (Figure S6B), from 5.6 Å to 4.7 Å, but the E325-R302 salt-bridge was only fully formed upon relaxing the occluded structure in the *Occ* equilibrium simulation (Figure S6C). The minimum distance between the carboxylate oxygen atoms of E325 and the nitrogen atoms of R302 was found to have an average value of 3.3±0.5 Å in *Occ*.

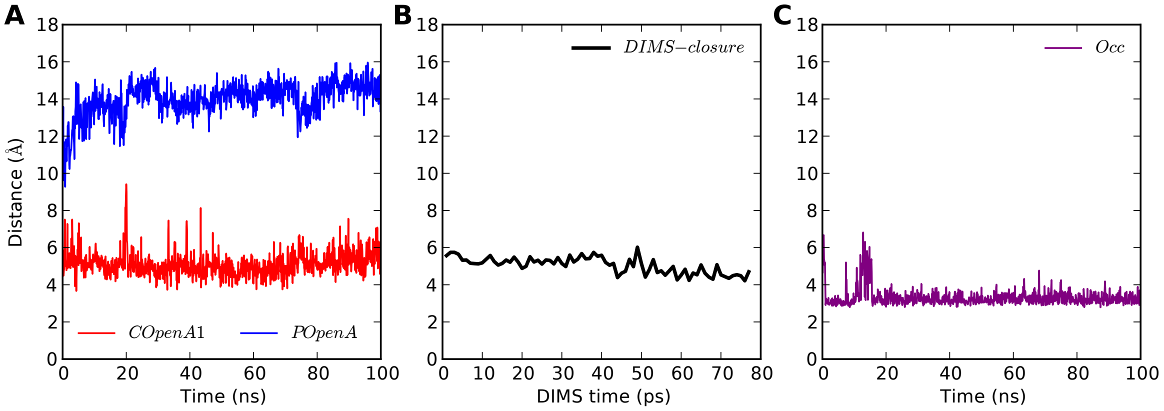

Figure S6: Salt-bridge E325-R302: minimum oxygen-nitrogen distance in *COpenA1*, *POpenA*, *Occ* and *DIMS-closure*. The minimum distance between the two oxygen atoms of the carboxylate group and the three nitrogen atoms of the arginine side-chains over the course of the simulations is plotted for *COpenA1* (red) and *POpenA* (blue) in **A,** for *DIMS closure* (black) in **B,** and *Occ* (purple) in **C**.

Additional analysis of DEER distances

Figure S7A shows analysis of DEER distances that were only present in the simulations based on the crystal structure (labelled residues 73–401, 136–401, and 137–401); the FucP-based homology model of periplasmic open LacY[19] misses part of the C-terminus including residue 401 so that we could not compute DEER distance distributions for simulations based on the model (*POpenA*, *Occ*). The computed distributions from the cytoplasmic open simulation agree well with the locations of the primary peaks from the experiments with the non-binding sugar NPGlc, which shifts the conformational equilibrium of LacY towards the cytoplasmic open state[10]. When the same distances are compared to experimental data with the binding sugar NPGal, which shifts the equilibrium away from the cytoplasmic open state[10], we observe that the computed peaks now agree with secondary or tertiary experimental peaks (Figure S7B).

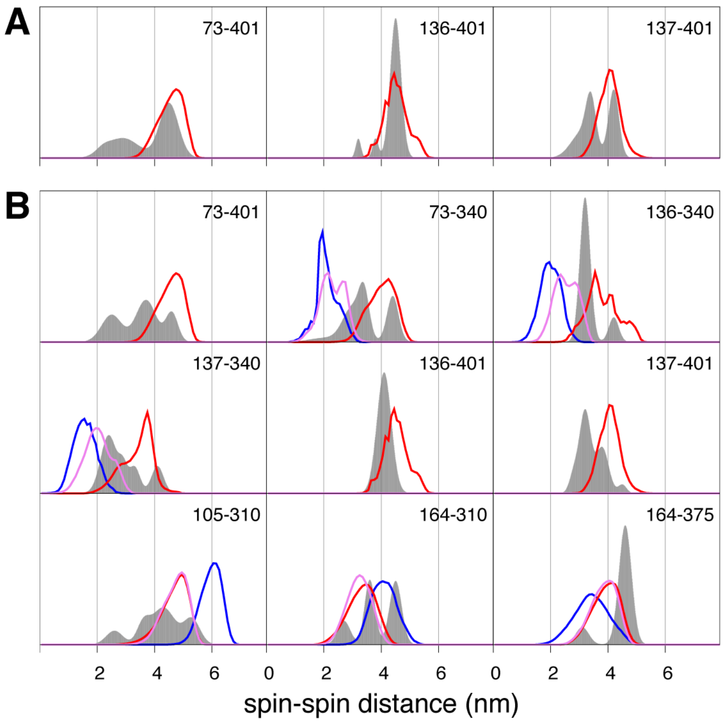

Figure S7: Comparison of DEER distance distributions from simulations and experiments. Experimental distributions are shown in gray. Distance distributions from simulations of cytoplasmic and periplasmic open as well as occluded LacY are indicated by red, blue and magenta lines. (**A**) Comparing additional residues pairs (73-401, 136-401 and 137-401) to the experimental distributions of predominately cytoplasmic open LacY. Experiments were in presence of non-binding sugar NPGlc[10]. For some distances 73-401, 136-401 and 137-401, only the simulations of cytoplasmic open LacY can be compared to the experiments. The homology model of the periplasmic open state and the model for the occluded state derived from DIMS MD are missing some residues at the extreme C-terminus such as residue 401. (**B**) Comparing simulations to the DEER distributions of predominately periplasmic open LacY. Experiments were performed in presence of binding sugar NPGal that shifts the conformational equilibrium[10].

Comparisons of DEER distances from the occluded and periplasmic open simulation with the experimental distributions from the experiments with NPGal (Figure S7B) generally exhibit the expected pattern where calculated peaks of distances on the cytoplasmic side (involving residues 73, 136, 137) are shifted towards smaller distances, indicating cytoplasmic gate closure. The calculated peak centers co-locate in the vicinity of the highest experimental peaks because NPGal shifts the conformational equilibrium towards the periplasmic open (and the occluded) state where the cytoplasmic gate is closed. Similarly, calculated distributions from the occluded and cytoplasmic open

simulations coincide on the periplasmic side (distances involving residues 105 and 164) although those peaks agree less well with the experimental data. As discussed in the main text, the distance 164–375 (on the periplasmic side) suffers from an unexpected local conformational change in the simulation *POpenA* that brings helices 5 and 11 closer together and thus leads to a distance that is smaller in the periplasmic open state than in the cytoplasmic open one.

Order parameter analysis of the equilibrium simulations

As discussed in the main text, we derived geometric order parameters to compare our simulations with crystal structures across the MFS transporter family. The order parameters were defined via the shortest Cα-Cα distance between the helices constituting the cytoplasmic or the periplasmic gate. The helix definitions used in the analysis are listed in Table S2.

Table S2. Gate helix definitions of MFS transporters with known structure.

<table>
<tr><td>Transporter</td><td>Structure
(PDB or
PMDB id)</td><td>TM4
(cytoplasmic gate)</td><td>TM10
(cytoplasmic gate)</td><td>TM1
(periplasmic gate)</td><td>TM 7
(periplasmic gate)</td></tr>
<tr><td>EmrD</td><td>2GFP:A</td><td>100-130</td><td>296-316</td><td>9-36</td><td>202-230</td></tr>
<tr><td>LacY</td><td>2V8N:A
PM0077183
PM0076824</td><td>104-136</td><td>312-340</td><td>7-35</td><td>220-250</td></tr>
<tr><td>NarU</td><td>4IU8:A</td><td>128-153</td><td>345-386</td><td>21-59</td><td>255-282</td></tr>
<tr><td>PepT$_{So}$</td><td>2XUT:A</td><td>109-137</td><td>405-434</td><td>15-41</td><td>292-328</td></tr>
<tr><td>PiPT</td><td>4J05:A</td><td>128-161</td><td>412-441</td><td>31-63</td><td>307-342</td></tr>
<tr><td>XylE</td><td>4GBY</td><td>122-153</td><td>370-398</td><td>8-46</td><td>277-307</td></tr>
<tr><td>YajR</td><td>3WDO</td><td>101-127</td><td>302-330</td><td colspan="2" rowspan="2">p. gate defined via sequence alignment with cytoplasmic open LacY</td></tr>
<tr><td>FucP</td><td>3O7Q</td><td>117-144</td><td>347-373</td></tr>
<tr><td>GlpT</td><td>1PW4</td><td colspan="2" rowspan="4">c. gate defined via structure alignment with periplasmic open LacY</td><td>20-54</td><td>253-279</td></tr>
<tr><td>PepT$_{St}$</td><td>4APS</td><td>13-46</td><td>284-314</td></tr>
<tr><td>POT</td><td>4IKV</td><td>23-51</td><td>280-323</td></tr>
<tr><td>NarK</td><td>4JR9:A</td><td>23-65</td><td>257-284</td></tr>
</table>

Our choice for the cytoplasmic and periplasmic order parameters agrees with previous studies of the gating mechanism of MFS transporters. The order parameter for the periplasmic gate of LacY was defined as the Cα distance between I32 and N245 on helices 1 and 7. Cross linking experiments based on computer simulations[21] implicated N245 and nearby I40 (in the helix 1–2 linker) in periplasmic gating[22]. The state of the cytoplasmic gate of LacY was tracked using the Cα distance between E126 and C333 on helices 4 and 10. These are close to residues F140, F334 and Y350, on helices 5, 10 and 11, respectively, which are conserved and have been implicated in cytoplasmic closure[23]. In addition our order parameter for the periplasmic gate (S32-Y298) of XylE agrees with previous work. Y298 is thought to prevent D-xylose permeation[24].

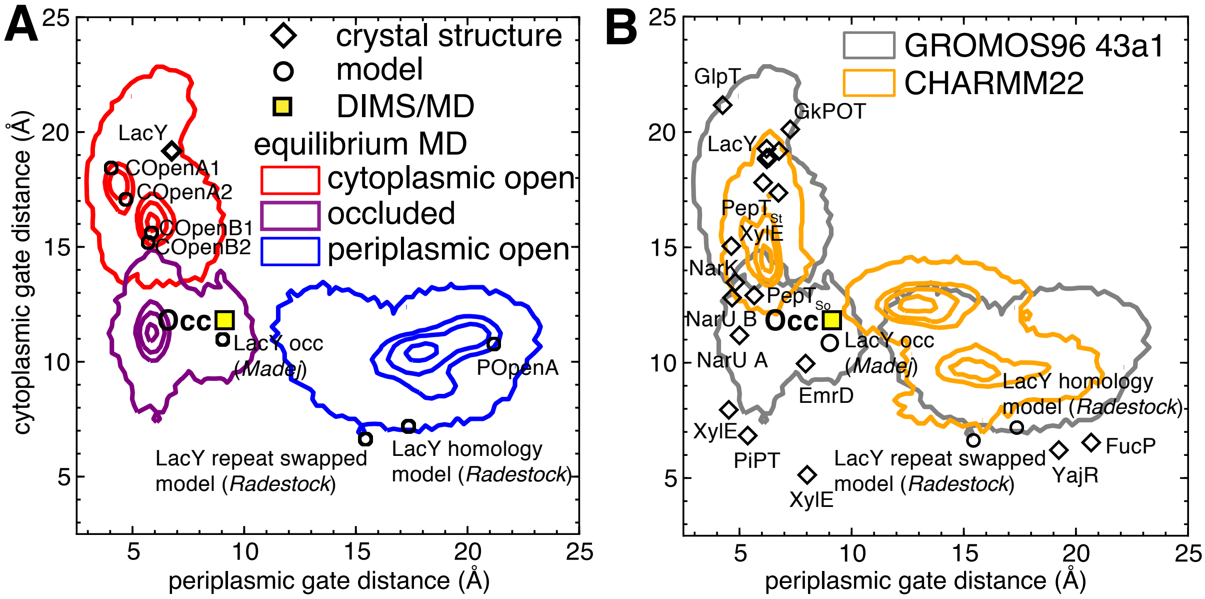

Figure S8: Order parameters for the equilibrium trajectories. In **A** the GROMOS trajectories are analyzed in more detail. The most populated regions in order parameter space are indicated by contour lines shown at 20%, 40% and 80% of the maximum bin occupancy. The order parameters for the final frames of the simulations with the GROMOS force field (*COpenA1*, *COpenA2*, *COpenB2, Occ* and *POpenA*) are depicted. The order parameter space visited in simulations started with cytoplasmic and periplasmic open as well as occluded structures are highlighted in red, blue and purple. Simulations with the CHARMM force field (**B**) are outlined in orange while the GROMOS trajectories from Figure 6 and Figure S8A are shown in gray. Experimental crystal structures are indicated as diamonds. See Figure 6 in the main text for further details.

The order parameter analysis for the simulations with cytoplasmic and periplasmic open LacY agrees with the functional characterisation based on water densities and pore profiles. In the equilibrium MD simulations of cytoplasmic open LacY the cytoplasmic order parameter, ~19 Å for the crystal structure, decreased in value, indicating closure of the cytoplasmic gate (as shown by the pore radius calculations), but not far enough to suggest a fully closed gate (as evidenced by the water densities). The periplasmic gating distances were mostly < 8 Å and so the periplasmic gate remained closed during the course of the simulations as indicated by the contour lines in Figure S8A. The order parameters for the simulations of periplasmic open LacY showed that it remained in this state. Limited periplasmic closure was detected for simulation *POpenB* (conducted with the CHARMM force field) (Figure S8B) which agreed with the pore radius analysis.

Equilibrium simulations using an alternative force field (CHARMM; orange lines in Figure S8B) showed very similar order parameter values to the GROMOS96 simulations discussed in the main text and Figure 6. The overlap in the order parameter regions in Figure S8B indicates that the observed conformational flexibility in LacY is a robust output from the simulations as it is not strongly dependent on the force field.

The order parameters also enable a more detailed analysis of the initial closure of the cytoplasmic open gate in simulations of cytoplasmic open LacY. Structures with a significantly closed cytoplasmic gate (~16 Å as opposed to ~19 Å) make up the most populated region in the 2D order parameter space in simulations with the GROMOS force field (Figure S8A), as shown by the contour lines. Closed conformations are even more

populated in the simulations with the CHARMM force field (Figure S8B). The order parameters also reveal differences between the simulations in respect to the initial closure of the cytoplasmic gate. Simulations *COpenA1* and *COpenA2* show a less drastic closure than *COpenB1*, *COpenB2* and *COpenC*, as judged by both their average (See Table S3) and their final structures which are highlighted for the GROMOS simulations in Figure S8A. The relatively high order parameter for the final frame for *COpenA1* illuminates the result from Poisson-Boltzmann electrostatics calculation for this frame. While the minimum pore radius dropped to ~1 Å in *COpenA1*, a value consistent with an functionally occluded structure, the cytoplasmic gate itself remained open and proton permeation appeared possible as shown by absence of a significant electrostatic free energy barrier (Figure S5). The water densities calculated from this trajectory also show that no functionally occluded structure was formed (see Figure 3D in the main text).

Table S3 Characterizing the initial closure of the cytoplasmic gate

	2V8N-crystal structure	*COpenA1*	*CopenA2*	*COpenB1*	*COpenB2*	*COpenC*
**Average/standard deviation** cytoplasmic order parameter (Å)	—	17.3±1.2	17.8±0.8	16.4±0.9	16.1±0.6	15.1±1.1
Cytoplasmic order parameter for the **final frame** (Å)	19.2	18.4	17.1	15.2	15.6	13.4

Equilibrium simulation with implicit solvent

Using an implicit solvent model, GBSW, greatly speeded up the DIMS simulations enabling us to track large-scale conformational changes. The GBSW model captures the salient features of a lipid bilayer. The membrane is modelled with a low dielectric core (ε=1; see also Figure 2 in the main text) and a head-group region where the dielectric constant starts to approach that of the surrounding aqueous solvent (ε=80). It is however clear that the model cannot reproduce the stabilizing water-protein interactions in internal protein cavities inside the membrane. To evaluate this limitation, we have run short equilibrium simulations, on the order of 10 ns, for the cytoplasmic open crystal structure (*COpen-GBSW*), the periplasmic open homology model (*POpen-GBSW*) as well as the occluded model derived from DIMS and atomistic equilibrium MD simulations (*Occ-GBSW*). The Cα RMSDs to their respective starting structure rose to ~4A for the simulation of cytoplasmic open (*COpen-GBSW;* Figure S9A) LacY; to ~2 A for the occluded transporter(*Occ-GBSW*; Figure S9B); and to 4.5 Å for the periplasmic open model (*POpen-GBSW*; Figure S9C). Taken together, the conformational drift is comparable to the atomistic equilibrium MD simulations. The occluded structure remained more similar to the final frame of *Occ*, that is, to the occluded structure relaxed in an atomistic lipid bilayer in equilibrium simulations, than to final frame of *DIMS-closure*, which was run with GBSW. The analysis suggests that simulations of

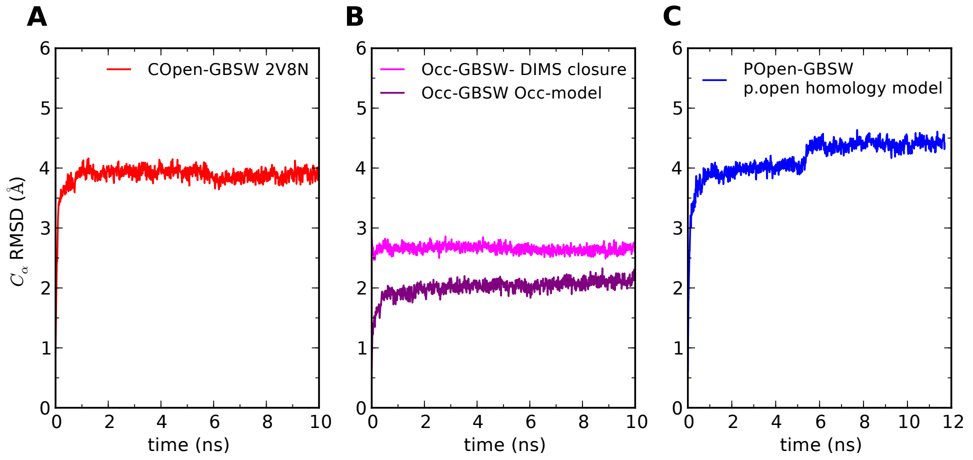

Figure S9: Cα RMSDs for equilbrium simulations with implicit solvent. The structural drift from the cytoplasmic open crystal structure 2V8N, the occluded model from DIMS and atomistic equilbrium simulations, and periplasmic open homology model in simulations *COpen-GBSW*, *Occ-GBSW* and *POpen-GBSW* is shown in **A**, **B** and **C** (in red, purple and blue) respectively. For comparison the Cα RMSD to the occluded final structure frame *DIMS-closure* is also shown in **B**. This struture had not been relaxed in an atomistic equilbrium MD simulation

cytoplasmic and periplasmic open as well as occluded LacY are reasonably stable in an implicit solvent and membrane environment.

Closed conformations of both the cytoplasmic and the periplasmic gates were favoured in the short GBSW simulations. The open gates of the cytoplasmic and the periplasmic open structures closed quickly in this set of simulations (Figure S10A, C). The order parameters dropped to values ~10 Å which puts the simulations into the region of the parameter plot populated by occluded conformations and goes beyond the initial closure of the cytoplasmic gate which has been repeatedly observed in simulations of cytoplasmic open LacY in an atomistic membrane environment, both in this study and in previous work. In addition, the gates of the occluded model became more closed in the GBSW simulations (Figure S10B). It is perhaps not surprising that gate closure is enhanced in absence of favourable water-protein interactions. The use of a continuum solvent model per-se may speed up gate closure as no water molecules have to move away as the gates close.

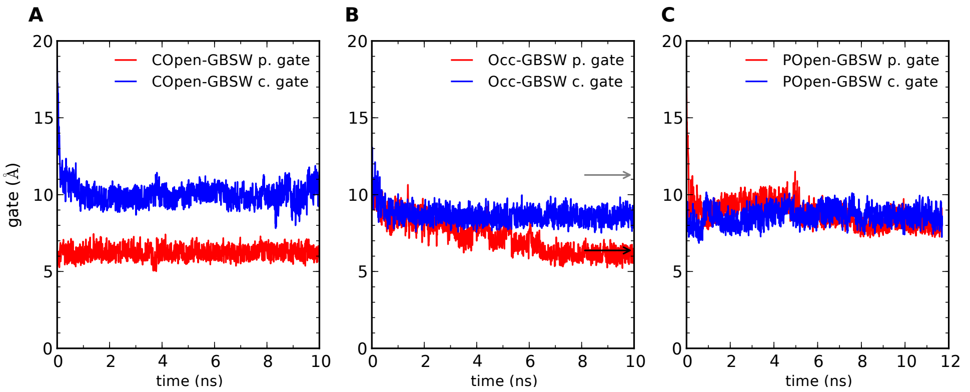

Figure S10: The order parameters for the cytoplasmic and periplasmic gates in the GBSW simulations. The order parameter for the cytoplasmic and the periplasmic gate are plotted in red and blue for simulations *COpen-GBSW* (**A**), *Occ-GBSW* (**B**) and *POpen-GBSW* (**C**). The average order parameters for the cytoplasmic and periplasmic gate in the atomistic equilibrium simulation Occ are indicated by grey and black arrows in **B**.

We used a soft-ratcheting algorithm to bias our DIMS simulations which will alleviate to some extent the neglect of water-protein interactions in the gates and the internal cavity of the transporter. Molecular dynamics steps that move the protein away from the target conformation, including moves that would lead to collapse of the internal cavity or unfolding, are likely to be rejected. In this way, the target structure itself provides an 'implicit' mean-field like potential that stabilizes the structure of the transporter and approximates all effects that contribute to the stability of the target conformation. Obtaining fully atomistic DIMS transitions between cytoplasmic and periplasmic open LacY will be an important future extension of this study, which will be greatly facilitated by the expected increases in available computer power.

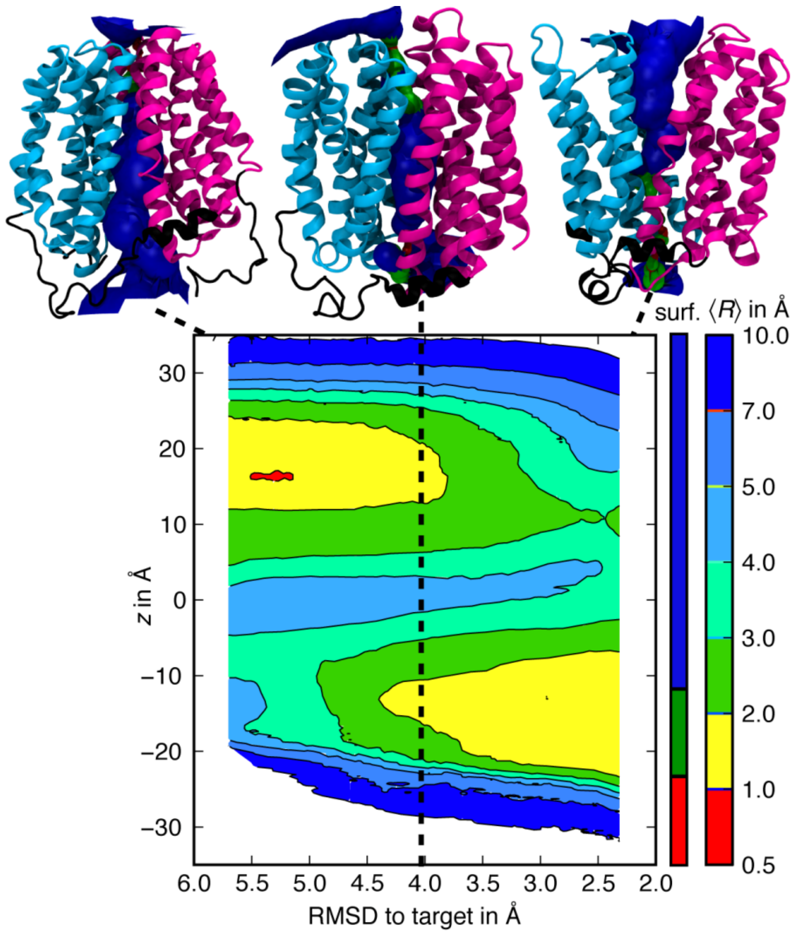

Figure S11: Average pore profiles for DIMS transitions between cytoplasmic and periplasmic open LacY. The cytoplasmic open starting, occluded DIMS intermediate, and the periplasmic open target structure are shown. The N-terminal (residues 7-185) and C-terminal (residues 220-399) domains are shown in cyan and magenta with the flexible linker connecting the domains in black. Pore surfaces calculated with HOLE[1] for cytoplasmic open, an occluded intermediate, and periplasmic open LacY are shown. Red, green and blue pore surfaces indicate pore positions with radii where no water (<1.15 Å), single file of water (between 1.15 Å and 2.3 Å) and multiple water molecules (>2.3 Å) could be accommodated, as specified by the left colour bar. Averaged pore profiles are plotted against the backbone RMSD distance of the N and C-terminal domains to the periplasmic open target structure. The average pore radius over the whole DIMS trajectory ensemble at a given *z* position and progress variable (backbone RMSD to target) is indicated by the coloured contours, specified by the right colour bar.

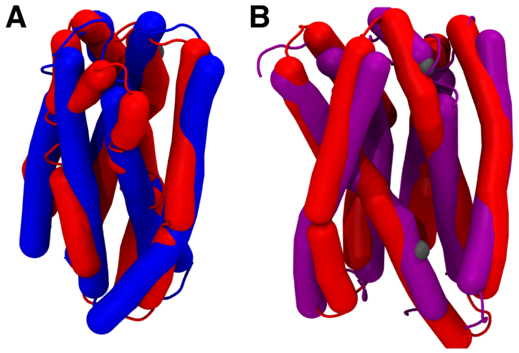

Figure S12: Structural comparison of the N-terminal domains of cytoplasmic and periplasmic open LacY (A) The Cα atoms of the N-terminal domains (residues 7 to 185) of cytoplasmic (red) and periplasmic open (blue) LacY were aligned using VMD[6] with a Cα RMSD of 3.6 Å. (**B**) The Cα atoms of the N-terminal domains (residues 7 to 185) of cytoplasmic and occluded LacY were aligned with a Cα RMSD of 3.2 Å. The transmembrane helices of the cytoplasmic open transporter are shown in red and the helices of the periplasmic open one in purple. The Cα atoms for I32 (at the top) and E126 (at the bottom) are shown in van der Waals representation (in grey) to indicate the location of the periplasmic and cytoplasmic gates. The transmembrane helices are depicted using Bendix[12].

References for Supplementary Information

1. Smart, O. S., Goodfellow, M. J. & Wallace, B. A. (1993). The pore dimensions of gramicidin A. *Biophys. J.* **65**, 2455-2460.
2. Abramson, J., Smirnova, I., Kasho, V., Verner, G., Kaback, H. R. & Iwata, S. (2003). Structure and mechanism of the lactose permease of Escherichia coli. *Science* **301**, 610-615.
3. Hermans, J., Berendsen, H. J. C., van Gunsteren, W. F. & Postma, J. P. M. (1984). A consistent empirical potential for water–protein interactions. *Biopolymers* **23**, 1513-1518.
4. van Gunsteren, W. F., Billeter, S. R., Eising, A. A., Hünenberger, P. H., Krüger, P., Mark, A. E., Scott, W. R. P. & Tironi, I. G. (1996). *Biomolecular Simulation: The GROMOS96 Manual and User Guide*, Vdf Hochschulverlag AG an der ETH Zürich, Zürich, Switzerland.
5. Beckstein, O., Tai, K. & Sansom, M. S. P. (2004). Not ions alone: barriers to ion permeation in nanopores and channels. *J. Am. Chem. Soc.* **126**, 14694-5.
6. Humphrey, W., Dalke, A. & Schulten, K. (1996). VMD: Visual molecular dynamics. *J. Mol. Graphics* **14**, 33-38.
7. Pettersen, E. F., Goddard, T. D., Huang, C. C., Couch, G. S., Greenblatt, D. M., Meng, E. C. & Ferrin, T. E. (2004). UCSF Chimera--a visualization system for exploratory research and analysis. *J. Comput. Chem.* **25**, 1605-12.
8. Goddard, T. D., Huang, C. C. & Ferrin, T. E. (2007). Visualizing density maps with UCSF Chimera. *J. Struct. Biol.* **157**, 281-7.
9. Laskowski, R. A., MacArthur, M. W., Moss, D. S. & Thornton, J. M. (1993). PROCHECK: a program to check the stereochemical quality of protein structures. *J. Appl. Cryst.* **26**, 283-291.
10. Smirnova, I., Kasho, V., Choe, J.-Y., Altenbach, C., Hubbell, W. L. & Kaback, H. R. (2007). Sugar binding induces an outward facing conformation of LacY. *Proc. Natl. Acad. Sci. U. S. A.* **104**, 16504-9.
11. Berger, O., Edholm, O. & Jähnig, F. (1997). Molecular dynamics simulations of a fluid bilayer of dipalmitoylphosphatidylcholine at full hydration, constant pressure, and constant temperature. *Biophys.~J.* **72**, 2002--2013.
12. Dahl, A. C. E., Chavent, M. & Sansom, M. S. P. (2012). Bendix: intuitive helix geometry analysis and abstraction. *Bioinformatics* **28**, 2193-2194.
13. Hess, B., Kutzner, C., van der Spoel, D. & Lindahl, E. (2008). GROMACS 4: Algorithms for Highly Efficient, Load-Balanced, and Scalable Molecular Simulation. *J. Chem. Theory Comput.* **4**, 435-447.
14. MacKerell, A. D., Bashford, D., Dunbrack, R. L., Evanseck, J. D., Field, M. J., Fischer, S., Gao, J., Guo, H., Ha, S., Joseph-McCarthy, D., Kuchnir, L., Kuczera, K., Lau, F. T. K., Mattos, C., Michnick, S., Ngo, T., Nguyen, D. T., Prodhom, B., Reiher, W. E., Roux, B., Schlenkrich, M., Smith, J. C., Stote, R., Straub, J., Watanabe, M., Wiórkiewicz-Kuczera, J., Yin, D. & Karplus, M. (1998). All-Atom Empirical Potential for Molecular Modeling and Dynamics Studies of Proteins *J. Phys. Chem. B* **102**, 3586-3616.
15. Klauda, J. B., Venable, R. M., Freites, J. A., O'Connor, J. W., Tobias, D. J., Mondragon-Ramirez, C., Vorobyov, I., MacKerell, A. D. & Pastor, R. W. (2010). Update of the CHARMM all-atom additive force field for lipids: validation on six lipid types. *J. Phys. Chem. B* **114**, 7830-43.
16. Guan, L., Mirza, O., Verner, G., Iwata, S. & Kaback, H. R. (2007). Structural determination of wild-type lactose permease. *Proc. Natl. Acad. Sci. U. S. A.* **104**, 15294-8.
17. Dang, S., Sun, L., Huang, Y., Lu, F., Liu, Y., Gong, H., Wang, J. & Yan, N. (2010). Structure of a fucose transporter in an outward-open conformation. *Nature* **467**, 734-738.

18. Prlić, A., Bliven, S., Rose, P. W., Bluhm, W. F., Bizon, C., Godzik, A. & Bourne, P. E. (2010). Pre-calculated protein structure alignments at the RCSB PDB website. *Bioinformatics* **26**, 2983-5.
19. Radestock, S. & Forrest, L. R. (2011). The alternating-access mechanism of MFS transporters arises from inverted-topology repeats. *J. Mol. Biol.* **407**, 698--715.
20. Chen, V. B., Arendall, W. B., III, Headd, J. J., Keedy, D. A., Immormino, R. M., Kapral, G. J., Murray, L. W., Richardson, J. S. & Richardson, D. C. (2010). MolProbity: all-atom structure validation for macromolecular crystallography. *Acta Crystallogr., Sect. D: Biol. Crystallogr.* **66**, 12-21.
21. Jensen, M. Ø., Yin, Y., Tajkhorshid, E. & Schulten, K. (2007). Sugar Transport across Lactose Permease Probed by Steered Molecular Dynamics. *Biophys. J.* **93**, 92-102.
22. Zhou, Y., Nie, Y. & Kaback, H. R. (2009). Residues Gating the Periplasmic Pathway of LacY. *Journal of Molecular Biology* **394**, 219-225.
23. Kasho, V. N., Smirnova, I. N. & Kaback, H. R. (2006). Sequence Alignment and Homology Threading Reveals Prokaryotic and Eukaryotic Proteins Similar to Lactose Permease. *J. Mol. Biol.* **358**, 1060-1070.
24. Sun, L., Zeng, X., Yan, C., Sun, X., Gong, X., Rao, Y. & Yan, N. (2012). Crystal structure of a bacterial homologue of glucose transporters GLUT1-4. *Nature* **490**, 361-366.
